# Supplementary material for: Genetic recombination and diversity of sapovirus in pediatric patients with acute gastroenteritis in Thailand, 2010–2018
Source: PeerJ. 2020 Feb 6;8:e8520. doi: 10.7717/peerj.8520 (PMC7007980; doi:10.7717/peerj.8520)
Supplement: Supplemental Information 2 [file peerj-08-8520-s002.docx]

RdRp sequences

>CMH-N018-10

AGATTTGCTGAACCCCATCCAGTTGTGTCTTGTGCCATTGAGGCCCTTTCTTCCCCTGCA

GAGGGCTACGTCAATGACATCAAGTTTGTGACACGTGGTGGGCTACCATCTGGGATGCCA

TTTACATCTGTTGTCAATTCCATCAACCACATGATATATGTGGCGGCAGCCATCTTGCAG

GCATACGAGAGCCACAATGTCCCATACACTGGGAATGTCTTCCAGGTGGAGACCATTCAC

ACGTATGGTGATGATTGCATGTACAGTGTGTGCCCTGCCACTGCATCAATCTTCCACACT

GTGCTCGCCAACCTGACATCATACGGACTGAAGCCCACAGCAGCTGACAAGAGTGATGCA

ATCAAACCAACCAATACACCAGTGTTTCTGAAGAGGACATTCACGCAGACCCCACATGGA

GTTCGTGCACTACTAGACATCACTTCCATAACTAGGCAGTTTTACTGGCTGAAAGCAAAC

AGAACATCTGACCCCTCTAGCCCACCTGCTTTTGATCGTCAGGCACGCAGTGCACAATTG

GAGAATGCACTGGCCTACGCTTCACAACACGGGCCTGTCATGTTTGACACCGTGCGCCAA

ATTGCCATAAAGACTGCTCAAGGGGAGGGACTGGTGCTTGTCAACACCAACTATGACCAG

GCTCTCGCCACCTACAATGCTTGGTTCATAGGTGGTACAGTACCTGACCCAGTGGGTTAC

ACTGAAGGAACCCACAAAATAGTGT

>CMH-S050-10

AGATTTGCTGAACCCCATCCAATTGTGTCTTGTGCTATTGAGGCCCTTTCTTCCCCTGCA

GAGGGCTATGTCAACGATATCAAGTTTGTGACACGTGGTGGTCTACCCTCTGGGATGCCA

TTTACATCTGTCGTCAATTCCATCAATCACATGATATATGTGGCGGCAGCCATTCTGCAG

GCATATGAAAGCCATAATGTCCCATACACTGGGAATGTTTTCCAGGTGGAGACCATCCAC

ACGTACGGTGATGACTGCATGTACAGTGTGTGCCCTGCCACCGCATCAATTTTCCACACT

GTGCTTGCCAACCTGACATCGTATGGACTGAAGCCCACTGCAGCTGACAAGAGTGATGCA

ATCAAACCAACCAACACACCAGTGTTTCTAAAGAGGACATTCACACAAACCCCACATGGA

GTCCGTGCACTACTAGACATCACTTCCATAACTAGACAGTTTTACTGGCTAAAAGCAAAT

AGAACATCTGACCCCTCTAGTCCACCTGCTTTTGATCGTCAGGCACGCAGTGCGCAACTG

GAGAACGCATTGGCCTATGCCTCACAACACGGGCCTGTCACGTTTGACACCGTGCGCCAA

ATTGCCATTAAGACTGCCCAAGGAGAGGGATTGGTGCTTGTAAATACCAACTATGACCAG

GCTCTCGCCACCTACAATGCTTGGTTCATAGGTGGTACAGTACCTGACCCAGTGGGTCAC

ACTGAAGGAACCCACAAAATAGTGT

>CMH-N131-13

AGATTTGCCGAACCCCATCCAATTGTGTCCTGTGCTATTGAGGCCCTTTCTTCCCCTGCA

GAGGGCTATGTCAACGATATCAAGTTTGTGACACGTGGTGGTCTACCCTCTGGGATGCCA

TTTACGTCTGTCGTCAATTCCATCAATCACATGATATATGTGGCGGCAGCCATTCTGCAG

GCATATGAAAGCCACAACGTCCCATACACTGGGAATGTTTTCCAGGTGGAGACCATTCAC

ACGTACGGTGATGACTGCATGTACAGTGTGTGCCCCGCCACCGCATCAATTTTCCACACT

GTGCTTGCCAACCTGACATCATATGGACTGAAGCCCACAGCAGCTGACAAAAGTGATGCA

ATCAAACCAACCAACACACCAGTGTTTCTAAAGAGGACATTCACACAAACTCCACATGGA

GTCCGTGCACTACTAGACATCACTTCTATAACTAGACAGTTTTACTGGCTAAAAGCAAAT

AGAACATCTGACCCCTCTAGTCCACCTGCTTTTGATCGTCAGGCACGCAGTGCACAATTG

GAGAACGCACTAGCCTATGCCTCACAACACGGGCCCGTCACGTTTGACACCGTGCGCCAA

ATTGCCATTAAGACTGCCCAAGGAGAGGGATTGGTGCTTGTTAACACCAACTATGACCAG

GCTCTCGCCACCTACAATGCTTGGTTCATAGGTGGTACAGTACCTGACCCAGCGGGTCAC

ACTGAAGGAACCCACAAAATAGTGT

>CMH-S004-13

AGATTTGCTGAACCCCATCCAATTGTGTCCTGTGCTATTGAGGCCCTTTCTTCCCCTGCA

GAGGGCTATGTCAACGATATCAAGTTTGTGACACGTGGTGGTCTACCCTCTGGGATGCCA

TTTACATCTGTCGTCAATTCCATCAATCACATGATATATGTGGCGGCAGCTATTCTGCAG

GCATATGAAAGCCACAACGTCCCATACACTGGGAATGTTTTCCAGGTGGAGACCATTCAC

ACGTACGGTGATGACTGCATGTACAGTGTGTGCCCCGCCACCGCATCAATTTTCCACACT

GTGCTTGCCAACCTGACATCATATGGACTGAAGCCCACAGCAGCTGACAAAAGTGATGCA

ATCAAACCAACCAACACACCAGTGTTTCTAAAGAGGACATTCACACAAACTCCACATGGA

GTCCGTGCACTACTAGACATCACTTCTATAACTAGACAGTTTTACTGGCTAAAAGCAAAT

AGAACATCTGACCCCTCTAGTCCACCTGCTTTTGATCGTCAGGCACGCAGTGCACAATTG

GAGAACGCACTAGCCTATGCCTCACAGCACGGGCCCGTCACGTTTGACACCGTGCGCCAA

ATTGCCATTAAGACTGCCCAAGGAGAGGGATTGGTGCTTGTTAACACCAACTATGACCAG

GCTCTCGCCACCTACAATGCTTGGTTCATAGGTGGTACAGTACCTGACCCAGCGGGCCAC

ACTGAAGGAACCCACAAAATAGTGT

>CMH-N006-15

AGATTTGCTGAACCCCATCCAATTGTGTCCTGTGCTATTGAGGCCCTTTCTTCCCCTGCA

GAGGGCTATGTCAACGATATCAAGTTTGTGACACGTGGTGGTTTACCCTCCGGGATGCCA

TTTACATCTGTCGTCAATTCCATCAATCACATGATATATGTGGCGGCAGCCATTCTGCAG

GCATATGAGAGCCACAATGTCCCATACACTGGGAATGTTTTCCAGGTGGAGACCATTCAT

ACGTACGGTGATGACTGCATGTACAGTGTGTGCCCTGCCACCGCATCAATTTTCCACACT

GTACTTGCCAACCTAACATCGTATGGACTGAAGCCCACAGCAGCTGACAAGAGTGACGCA

ATCAAACCAACCAACACACCAGTGTTTCTGAAGAGGACATTCACGCAAACCCCACATGGA

GTCCGTGCACTACTAGACATCACTTCCATAACTAGACAGTTTTACTGGCTGAAAGCAAAT

AGAACATCTGACCCCTCTAGTCCACCTGCTTTTGATCGTCAGGCACGCAGTGCACAACTG

GAGAACGCACTAGCCTATGCCTCACAACACGGACCTGTCACGTTTGACACCGTGCGCCAA

ATTGCCATCAAGACTGCCCAAGGAGAGGGACTGGTGCTTGTTAACACCAACTATGACCAG

GCTCTCGCCACCTACAATGCTTGGTTCATAGGTGGTACAGTACCTGACCCAGTGGGTCAC

ACTGAAGGGACCCACAAAATAGTGT

>CMH-N007-15

AGATTTGCTGAACCCCATCCAATTGTGTCCTGTGCTATTGAGGCCCTTTCTTCCCCTGCA

GAGGGCTATGTCAACGATATCAAGTTTGTGACACGTGGTGGTTTACCCTCCGGGATGCCA

TTTACATCTGTCGTCAATTCCATCAATCACATGATATATGTGGCGGCAGCCATTCTGCAG

GCATATGAGAGCCACAATGTCCCATACACTGGGAATGTTTTCCAGGTGGAGACCATTCAT

ACGTACGGTGATGACTGCATGTACAGTGTGTGCCCTGCCACCGCATCAATTTTCCACACT

GTACTTGCCAACCTAACATCGTATGGACTGAAGCCCACAGCAGCTGACAAGAGTGACGCA

ATCAAACCAACCAACACACCAGTGTTTCTGAAGAGGACATTCACGCAAACCCCACATGGA

GTCCGTGCACTACTAGACATCACTTCCATAACTAGACAGTTTTACTGGCTGAAAGCAAAT

AGAACATCTGACCCCTCTAGTCCACCTGCTTTTGATCGTCAGGCACGCAGTGCACAACTG

GAGAACGCACTAGCCTATGCCTCACAACACGGACCTGTCACGTTTGACACCGTGCGCCAA

ATTGCCATCAAGACTGCCCAAGGAGAGGGACTGGTGCTTGTTAACACCAACTATGACCAG

GCTCTCGCCACCTACAATGCTTGGTTCATAGGTGGTACAGTACCTGACCCAGTGGGTCAC

ACTGAAGGGACCCACAAAATAGTGT

>CMH-S166-15

AGATTTGCTGAACCCCATCCAATTGTGTCCTGTGCTATTGAGGCCCTTTCTTCCCCTGCA

GAGGGCTATGTCAACGATATCAAGTTTGTGACACGTGGTGGTTTACCCTCCGGGATGCCA

TTTACATCTGTCGTCAATTCCATCAATCACATGATATATGTGGCGGCAGCCATTCTGCAG

GCATATGAGAGCCACAATGTCCCATACACTGGGAATGTTTTCCAGGTGGAGACCATTCAT

ACGTACGGTGATGACTGCATGTACAGTGTGTGCCCTGCCACCGCATCAATTTTCCACACT

GTACTTGCCAACCTAACATCGTATGGACTGAAGCCCACAGCAGCTGACAAGAGTGACGCA

ATCAAACCAACCAACACACCAGTGTTTCTGAAGAGGACATTCACGCAAACCCCACATGGA

GTCCGTGCACTACTAGACATCACTTCCATAACTAGACAGTTTTACTGGCTGAAAGCAAAT

AGAACATCTGACCCCTCTAGTCCACCTGCTTTTGATCGTCAGGCACGCAGTGCACAACTG

GAGAACGCACTAGCCTATGCCTCACAACACGGACCTGTCACGTTTGACACCGTGCGCCAA

ATTGCCATCAAGACTGCCCAAGGAGAGGGACTGGTGCTTGTTAACACCAACTATGACCAG

GCTCTCGCCACCTACAATGCTTGGTTCATAGGTGGTACAGTACCTGACCCAGTGGGTCAC

ACTGAAGGGACCCACAAAATAGTGT

>CMH-S198-16

AGATTTGCTGAGCCCCACCCAATCGTGTCTTGTGCCATTGAGGCGCTTTCTTCCCCTGCA

GAGGGCTACGTGAATGACATCAAGTTCGTGACACGCGGCGGTCTACCATCCGGGATGCCA

TTCACATCTGTTGTCAACTCCATCAACCATATGATATATGTAGCGGCAGCCATTCTGCAG

GCGTACGAAAGCCACAGTGTTCCGTACACTGGAAATGTTTTCCAAGTGGAAACAGTCCAC

ACGTATGGCGATGATTGCATGTACAGTGTGTGCCCTGCTACTGCATCAATTTTCCACACT

GTGCTTGCCAACCTAACATCGTATGGACTTAAGCCCACTGCAGCAGACAAGAGTGAAGCA

ATCAAGCCAACCAACACGCCAGTGTTTTTGAAGAGGACATTCACACAAACCCCGCATGGA

GTCCGAGCACTGCTAGACATCACTTCTATAACTAGACAGTTTTACTGGCTGAAGGCTAAT

AGGACATCAGACCCTTCTAGCCCACCTGCTTTCGATCGACAAGCACGCAGTGCGCAACTG

GAGAACGCGCTAGCCTATGCTTCACAACATGGACCTGTTGTGTTTGACACCGTGCGCCAA

ATTGCCATAAAGACTGCCCAAGGGGAGGGATTGGTGCTTGTCAACACCAATTATGACCAG

GCTCTCGCCACCTACAATGCTTGGTTCATAGGTGGTACAGTACCTGACCCAGTAGGTCAC

ACTGAAGGAACCCACAAATTAGTGT

>CMH-S229-16

AGATTTGCTGAGCCCCACCCAATCGTGTCTTGTGCCATTGAGGCGCTTTCTTCCCCTGCA

GAGGGCTACGTGAATGATATCAAGTTCGTGACACGCGGCGGTCTACCATCCGGGATGCCA

TTCACATCTGTTGTCAACTCCATCAACCATATGATATATGTAGCGGCAGCCATTCTGCAG

GCGTACGAAAGCCACAGTGTCCCGTACACTGGAAATGTTTTCCAAGTGGAAACAGTCCAC

ACGTATGGCGATGATTGCATGTACAGTGTGTGCCCTGCCACTGCATCAATTTTCCACACC

GTGCTTGCCAACCTAACATCGTATGGACTCAAGCCCACTGCAGCAGACAAGAGTGAAGCA

ATCAAGCCAACCAACACGCCAGTGTTTTTGAAGAGGACATTCACACAAACCCCGCATGGA

GTCCGAGCACTGCTAGACATCACTTCTATAACTAGACAGTTTTACTGGCTGAAGGCTAAT

AGGACATCGGACCCCTCTAGCCCACCTGCTTTCGATCGACAAGCACGCAGTGCGCAACTG

GAGAACGCGCTAGCTTATGCTTCACAACATGGACCTGTTGTGTTTGACACCGTGCGCCAA

ATTGCCATAAAGACTGCCCAAGGGGAGGGATTGGTGCTTGTCAACACCAATTATGACCAG

GCTCTCGCCACCTACAATGCTTGGTTCATAGGTGGTACAGTACCTGACCCAGTAGGTCAC

ACTGAAGGAACCCACAAAATAGTGT

>CMH-ST016-16

AGATTTGCTGAGCCCCACCCAATCGTGTCTTGTGCCATTGAGGCGCTTTCTTCCCCTGCA

GAGGGCTACGTGAATGACATCAAGTTCGTGACACGCGGCGGTCTACCATCCGGGATGCCA

TTCACATCTGTTGTCAACTCCATCAACCATATGATATATGTAGCGGCAGCCATTCTGCAG

GCGTACGAAAGCCACAGTGTCCCGTACACTGGAAATGTTTTCCAAGTGGAAACAGTCCAC

ACGTATGGCGATGATTGCATGTACAGTGTGTGCCCTGCCACTGCATCAATTTTCCACACT

GTGCTTGCCAACCTAACATCGTATGGACTCAAGCCCACTGCAGCAGACAAGAGTGAAGCA

ATCAAGCCAACCAACACGCCAGTGTTTTTGAAGAGGACATTCACACAAACCCCGCATGGA

GTCCGAGCACTGCTAGACATCACTTCTATAACTAGACAGTTTTACTGGCTGAAGGCTAAT

AGGACATCAGACCCTTCTAGCCCACCTGCTTTCGATCGACAAGCACGCAGTGCGCAACTG

GAGAACGCGCTAGCCTATGCTTCACAACATGGACCTGTTGTGTTTGACACCGTGCGCCAA

ATTGCCATAAAGACTGCCCAAGGGGAGGGATTGGTGCTTGTCAACACCAATTATGACCAG

GCTCTCGCCACCTACAATGCTTGGTTCATAGGTGGTACAGTACCTGACCCAGTAGGTCAC

ACTGAAGGAACCCACAAAATAGTGT

>CMH-ST090-16

AGATTTGCTGAGCCCCATCCAATTGTGTCCTGTGCTATTGAGGCCCTTTCTTCCCCTGCA

GAGGGCTATGTCAACGATATCAAGTTTGTGACACGTGGTGGTTTACCCTCTGGAATGCCA

TTTACATCTGTCGTCAATTCCATCAATCACATGATATATGTGGCGGCAGCCATTCTGCAG

GCATATGAGAGCCACAATGTCCCATACACTGGGAATGTTTTCCAGGTGGAGACCATTCAC

ACGTACGGTGATGACTGCATGTACAGTGTATGCCCTGCCACCGCATCAATTTTCCACACT

GTACTTGCCAACTTAACATCGTATGGACTGAAGCCCACAGCAGCTGACAAGAGTGATGCA

ATCAAACCAACCAACACACCAGTGTTTCTGAAGAGGACATTCACGCAAACCCCACATGGA

GTCCGTGCACTACTAGACATCACTTCCATAACTAGACAGTTTTACTGGCTGAAAGCAAAT

AGAACATCTGACCCCTCTAGTCCACCTGCTTTTGATCGTCAGGCACGCAGTGCACAACTG

GAGAACGCACTAGCCTATGCCTCACAACACGGACCTGTCACGTTTGACACTGTGCGCCAA

ATTGCCATCAAGACTGCCCAAGGAGAGGGACTGGTGCTTGTTAACACCAACTATGACCAG

GCTCTCGCCACCTACAATGCTTGGTTCATAGGTGGTACAGTACCTGACCCAGTGGGTCAC

ACTGAAGGAACCCACAAAATAGTGT

>CMH-ST163-16

AGATTTGCTGAGCCCCACCCAATCGTGTCTTGTGCCATTGAGGCGCTTTCTTCCCCTGCA

GAGGGCTACGTGAATGACATCAAGTTCGTGACACGCGGCGGTCTACCATCCGGGATGCCA

TTCACATCTGTTGTCAACTCCATCAACCATATGATATATGTAGCGGCAGCCATTCTGCAG

GCGTACGAAAGCCACAGTGTTCCGTACACTGGAAATGTTTTCCAAGTGGAAACAGTCCAC

ACGTATGGCGATGATTGCATGTACAGTGTGTGCCCTGCCACTGCATCAATTTTCCACACT

GTGCTTGCCAACCTAACATCGTATGGACTTAAGCCCACTGCAGCGGACAAGAGTGAAGCA

ATCAAGCCAACCAACACGCCAGTGTTTTTGAAGAGGACATTCACACAAACCCCGCATGGA

GTCCGAGCACTGCTAGACATCACTTCTATAACTAGACAGTTTTACTGGCTGAAGGCTAAT

AGGACATCAGACCCTTCTAGCCCACCTGCTTTCGATCGACAAGCACGCAGTGCGCAACTG

GAGAACGCGCTAGCCTATGCTTCACAACATGGACCTGTTGTGTTTGACACCGTGCGCCAA

ATTGCCATAAAGACTGCCCAAGGGGAGGGATTGGTGCTTGTCAACACCAATTATGACCAG

GCTCTCGCCACCTACAATGCTTGGTTCATAGGTGGTACAGTACCTGACCCAGTAGGTCAC

ACTGAAGGAACCCACAAAATAGTGT

>CMH-ST199-16

AGATTTGCTGAACCCCACCCAATTGTGTCCTGCGCCATTGAGGCCCTTTCTTCCCCTGCA

GAGGGTTATGTCAATGACATTAAGTTTGTGACACGTGGTGGTCTGCCGTCTGGGATGCCA

TTTACATCCGTCATCAATTCCATCAATCATATGATATACGTGGCGGCAGCCATTTTGCAG

GCATACGAAAGCCACAATGTCCCATACACCGGGAACGTTTTCCAGGTGGAGACCATTCAC

ACGTACGGTGATGACTGCATGTACAGTGTGTGCCCTGCCACCGCATCAATTTTCCACACT

GTGCTTGCCAACCTGACATCATATGGACTGAAGCCTACAGCAGCTGACAAGAGTGATGCA

ATCAAACCAACCAACACACCAGTGTTTCTGAAGAGGACATTCACACAAACCCCACATGGA

GTCCGTGCACTACTTGACATCACTTCTATAACTAGACAGTTTTACTGGCTAAAAGCAAAC

AGAACATCTGACCCCTCTAGCCCGCCTGCTTTTGACCGTCAAGCGCGTAGTGCACAACTG

GAGAACGCACTAGCCTATGCCTCACAACACGGGCCTGTCACGTTTGACACCGTGCGCCAA

ATTGCCATCAAGACTGCCCAAGGAGAGGGACTGGTGCTTGTTAACACCAACTATGACCAG

GCTCTCGCCACCTACAATGCTTGGTTCATAGGTGGTACAGTACCTGATCCAGTGGGTCAC

ACTGAAGGAACCCACAAAATAGTGT

>CMH-S003-17

AGATTTGCTGAGCCCCACCCAATCGTGTCTTGTGCCATTGAGGCGCTTTCTTCCCCTGCA

GAGGGCTACGTCAATGACATCAAGTTCGTGACACGCGGTGGTCTACCATCCGGGATGCCA

TTCACATCTGTTGTCAACTCCATCAACCATATGATATATGTAGCGGCAGCCATTCTGCAG

GCGTACGAAAGCCACAGTGTCCCGTACACTGGAAATGTTTTTCAAGTGGAAACAGTCCAC

ACGTATGGCGATGATTGCATGTACAGTGTGTGCCCTGCCACTGCATCAATTTTCCACACT

GTGCTTGCCAACCTAACATCGTATGGACTCAAACCCACTGCAGCAGACAAGAGTGAAGCA

ATCAAGCCAACCAACACGCCAGTGTTTTTGAAGAGGACATTCACACAAACCCCGCATGGA

GTCCGAGCACTGCTAGACATCACTTCTATAACTAGACAGTTTTACTGGCTGAAGGCTAAT

AGGACATCAGACCCTTCCAGCCCACCTGCTTTCGATCGACAAGCACGCAGTGCGCAACTG

GAGAACGCGCTAGCCTATGCTTCACAACATGGACCTGTTGTGTTCGACACCGTGCGCCAA

ATTGCCATAAAGACTGCCCAAGGGGAGGGATTGGTGCTTGTCAACACCAATTATGACCAG

GCTCTCGCCACCTACAATGCTTGGTTCATAGGTGGTACAGTACCTGACCCAGTAGGTCAC

ACTGAAGGAACCCACAAAATAGTGT

>CMH-S023-17

AGATTTGCTGAGCCCCATCCAATTGTGTCCTGTGCTATTGAGGCCCTTTCTTCCCCTGCA

GAGGGCTATGTCAACGATATCAAGTTTGTGACACGTGGTGGTTTACCCTCCGGAATGCCA

TTCACATCTGTCGTCAATTCCATCAACCACATGATATATGTGGCGGCAGCCATTCTACAG

GCATATGAGAGCCACAATGTTCCATACACTGGGAATGTTTTCCAGGTGGAGACCATTCAC

ACGTACGGTGATGACTGCATGTACAGTGTATGCCCTGCCACCGCATCAATTTTCCACACT

GTACTTGCCAACTTAACATCGTATGGACTGAAGCCCACAGCAGCTGACAAGAGTGATGCA

ATCAAACCAACCAACACACCAGTGTTTCTGAAGAGGACATTCACGCAAACCCCACATGGA

GTCCGTGCATTACTGGATATCACTTCCATAACTAGACAGTTTTACTGGCTGAAAGCAAAT

AGAACATCTGACCCCTCTAGTCCACCTGCTTTTGATCGTCAGGCACGCAGTGCACAACTG

GAGAACGCACTAGCCTATGCCTCACAACACGGACCTGTCACGTTTGACACTGTGCGCCAA

ATTGCCATCAGGACTGCCCAAGGAGAGGGACTGGTGCTTGTTAACACCAACTATGACCAG

GCTCTCGCCACCTACAATGCTTGGTTCATAGGTGGTACAGTACCTGACCCAGTGGGTCAC

ACTGAAGGAACCCACAAAATAGTGT

>CMH-S089-17

AGATTTGCTGAACCCCATCCAATTGTGTCCTGTGCTATTGAGGCCCTTTCATCCCCTGCA

GAGGGCTATGTCAACGACATTAAGTTTGTGACACGTGGTGGTCTACCCTCCGGGATGCCA

TTTACATCTGTCGTCAATTCCATCAATCACATGATATATGTGGCGGCAGCCATTTTGCAG

GCATATGAGAGCCACAATGTCCCATACACTGGGAATGTTTTCCAGGTGGAGACCATTCAT

ACGTACGGTGATGACTGTATGTACAGTGTGTGCCCTGCCACCGCGTCAATTTTCCACACT

GTACTTGCCAACCTAACATCGTATGGACTGAAGCCCACAGCAGCTGACAAGAGTGATGCA

ATTAAACCAACCAACACACCAGTGTTTCTGAAGAGGACATTCACGCAAACCCCACATGGA

GTCCGTGCACTACTAGACATCACTTCCATAACTAGACAGTTTTACTGGCTCAAAGCAAAT

AGAACATCTGACCCCTCTAGTCCACCTGCTTTTGATCGTCAGGCACGCAGTGCACAACTG

GAGAACGCACTAGCCTATGCCTCACAACATGGACCTGTCACGTTTGACACCGTGCGCCAA

ATCGCCATCAAGACTGCCCAAGGAGAGGGACTGGTGCTTGTTAACACCAACTATGACCAG

GCTCTCGCCACCTACAATGCTTGGTTCATAGGTGGTACAGTACCTGACCCAGTGGGTCAC

ACTGAAGGAACCCACAAAATAGTGT

>CMH-R031-18

AGATTTGCTGAGCCCCACCCAATCGTATCTTGTGCCATTGAGGCGCTTTCTTCCCCTGCA

GAGGGCTACGTGAATGACATCAAGTTCGTGACACGCGGCGGTCTACCATCCGGGATGCCA

TTTACGTCTGTTGTTAACTCCATCAACCATATGATATATGTAGCGGCAGCCATTCTGCAG

GCGTACGAAAGCCACAGTGTCCCGTACACTGGAAATGTTTTCCAAGTGGAAACAGTCCAC

ACGTATGGCGATGATTGCATGTACAGTGTGTGCCCTGCCACTGCATCAATTTTCCACACT

GTGCTTGCCAACCTAACATCGTATGGACTCAAGCCCACTGCAGCAGACAAGAGTGAAGCA

ATCAAGCCAACCAACACGCCAGTGTTTTTGAAGAGGACATTCACACAAACCCCGCATGGA

GTCCGAGCACTGCTAGACATCACTTCTATAACTAGACAGTTTTACTGGCTGAAGGCTAAT

AGAACATCAGACCCTTCTAGCCCACCTGCTTTCGACCGACAAGCACGCAGTGCGCAACTG

GAGAACGCGCTAGCCTATGCTTCACAACATGGACCTGTTGTGTTTGACACCGTGCGCCAA

ATTGCCATAAAGACTGCCCAAGGGGAGGGATTGGTGCTTGTCAACACCAATTATGACCAG

GCTCTCGCCACCTACAATGCTTGGTTCATAGGTGGTACAGTACCTGACCCAGTAGGTCAC

ACTGAAGGAACCCACAAAATAGTGT

>CMH-S174-18

AGATTTGCTGAACCCCACCCAATTGTGTCCTGTGCTATTGAGGCCCTTTCTTCCCCTGCA

GAGGGATATGTCAACGATATCAAATTTGTGACACGTGGTGGTTTACCTTCTGGGATGCCA

TTCACATCTGTCGTCAATTCCATCAATCACATGATATATGTGGCGGCAGCTATTCTGCAG

GCATATGAGAGCCATAATGTCCCATACACTGGGAATGTTTTCCAAGTGGAGACCATTCAC

ACATACGGTGATGACTGCATGTACAGTGTGTGCCCTGCCACCGCATCAATTTTCCACACT

GTACTTGCTAACCTAACATCGTATGGACTGAAGCCCACAGCAGCTGACAAGAGTGATGCA

ATCAAACCAACCAACACACCAGTGTTTCTGAAGAGGACATTCACGCAAACCCCGCATGGT

ATCCGTGCACTACTAGACATCACTTCCATAACTAGACAGTTTTACTGGCTGAAAGCAAAT

AGAACATCTGACCCCTCTAGTCCACCTGCTTTTGATCGTCAGGCACGCAGTGCACAACTG

GAGAACGCACTAGCCTATGCCTCACAGCACGGACCTGTCACGTTCGACACCGTGCGCCAA

ATTGCCATCAAGACTGCCCAAGGAGAGGGACTGGTGCTTGTCAACACCAACTATGACCAG

GCACTCGCCACCTACAATGCTTGGTTCATAGGTGGTACAGTACCTGACCCAGTGGGTCAC

ACTGAAGGAACCCACAAAATAGTGT

>CMH-ST097-18

AGATTTGCTGAGCCCCACCCAATCGTATCTTGTGCCATTGAGGCGCTTTCTTCCCCTGCA

GAGGGCTACGTGAATGACATCAAGTTCGTGACACGCGGCGGTCTACCATCCGGGATGCCA

TTTACGTCTGTTGTTAACTCCATCAACCATATGATATATGTAGCGGCAGCCATTCTGCAG

GCGTACGAAAGCCACAGTGTCCCGTACACTGGAAATGTTTTCCAAGTGGAAACAGTCCAC

ACGTATGGCGATGATTGCATGTACAGTGTGTGCCCTGCCACTGCATCAATTTTCCACACT

GTGCTTGCCAACCTAACATCGTATGGACTCAAGCCCACTGCAGCAGACAAGAGTGAAGCA

ATCAAGCCAACCAACACGCCAGTGTTTTTGAAGAGGACATTCACACAAACCCCGCATGGA

GTCCGAGCACTGCTAGACATCACTTCTATAACTAGACAGTTTTACTGGCTGAAGGCTAAT

AGAACATCAGACCCTTCTAGCCCACCTGCTTTCGACCGACAAGCACGCAGTGCGCAACTG

GAGAACGCGCTAGCCTATGCTTCACAACATGGACCTGTTGTGTTTGACACCGTGCGCCAA

ATTGCCATAAAGACTGCCCAAGGGGAGGGATTGGTGCTTGTCAACACCAATTATGACCAG

GCTCTCGCCACCTACAATGCTTGGTTCATAGGTGGTACAGTACCTGACCCAGTAGGTCAC

ACTGAAGGAACCCACAAAATAGTGT

>CMH-ST169-18

AGATTTGCTGAACCCCACCCAATTGTGTCCTGTGCTATTGAGGCCCTTTCTTCCCCTGCA

GAGGGCTATGTCAACGATATCAAGTTTGTGACACGTGGTGGTTTACCCTCTGGGATGCCA

TTTACATCTGTCGTTAATTCCATCAATCACATGATATATGTGGCGGCAGCTATTCTGCAG

GCATATGAGAGCCACAATGTCCCATACACTGGGAATGTTTTCCAGGTGGAGACCATTCAC

ACATACGGTGATGACTGCATGTACAGTGTGTGCCCTGCCACTGCATCAATTTTCCACACT

GTACTTGCTAACCTAACCTCGTATGGACTGAAGCCCACAGCAGCTGACAAGAGTGATGCA

ATTAAACCAACCAACACACCAGTGTTTCTGAAGAGGACATTCACGCAAACCCCGCATGGT

GTCCGTGCACTACTAGACATCACTTCCATAACTAGACAGTTTTACTGGCTGAAAGCAAAT

AGAACATCTGACCCCTCTAGTCCACCTGCTTTTGATCGTCAGGCACGCAGTGCACAATTG

GAGAACGCACTAGCCTATGCCTCACAGCACGGACCTGTCACGTTCGACACCGTGCGCCAA

ATTGCCATCAAGACTGCCCAAGGAGAGGGACTGGTGCTTGTCAACACCAACTATGACCAG

GCTCTCGCCACCTACAATGCTTGGTTCATAGGTGGTACAGTACCTGACCCAGTGGGTCAC

ACTGAAGGAACCCACAAAATAGTGT

>CMH-S152-15

AGATTCATGGAAAGTAGCCCATTGGTGTCCTGTGCAATAGAGTCCCTGTCCTCACCAGCA

ATTGGCTATCTCAATGACATTAAATTTGTAACCAAAGGGGGTCTCCCATCAGGAATGCCC

TTCACCTCAGTCCTCAACTCGGTGAACCACATGATATACTTTGCAGCGGGTGTGCTCAAA

GCTTACGAGGACCACCACGTCCCATACACTGGCAATGTATTTCAGATAGAGACTGTCCAC

ACCTATGGTGATGACTGTATCTATAGTGTTTGTCCTGCCACCGCCTCTATCTTTGGTTCT

GTTCTCGCCAACCTGTCTTCCTTTGGTCTCAAGCCCACTGCCGCTGACAAAACTGCAGAA

ATCAAGCCCACCCAAACACCAGTTTTCCTGAAAAGAACATTCACACAGACGCCCTATGGG

GTGAGGGCACTATTGGACATCAATTCCATCATTCGGCAGTTCTACTGGGTTAAGGCGAAC

CGCACTAGTGACCCATCTAGTCCCCCTGCATTTGATCGCACTGCCCGCAGTGCCCAGTTG

GAAGCAGCCCTAGCCTATGCATCACAACATGGACCTTTAGTGTTTGACAAGGTGCGTGAT

ATTGCCATCAAAACGGCCGAAGGAGAGGGTGTGGTGCTTGTGAATACAAACTTTGATTTG

GCTCTCGCCACCTACAATGCCTGGTTCATAGGTGGTACAGCTCCAGATCCAGAGCGCCCC

ACTGAAGGTGCACCCAAATTAGTGT

>CMH-S252-15

AGATTCATGGAAAGTAGCCCATTGGTGTCTTGTGCAATAGAGTCCCTGTCCTCACCAGCA

ATTGGCTATCTCAATGACATCAAGTTTGTAACCAAAGGGGGTCTCCCATCAGGAATGCCC

TTCACCTCAGTTATCAACTCGGTGAACCACATGATATACTTTGCAGCGGGTGTGCTCAAA

GCTTATGAGGACCACCACGTCCCATACACTGGCAATGTATTTCAGATAGAGACTGTCCAC

ACCTATGGTGATGATTGTATTTATAGTGTTTGTCCTGCCACCGCTTCTATCTTTGGCTCT

GTTCTCGCCAACCTGTCCTCCTTCGGTCTCAAGCCCACTGCCGCTGACAAAACTGCAGAA

ATTAAGCCCACCCAAACACCAGTTTTCCTGAAAAGAACATTCACACAGACGCCCTATGGG

GTGAGGGCACTATTGGACATTAACTCCATCATTCGGCAGTTTTACTGGGTTAAGGCGAAT

CGCACTAGTGACCCATCTAGCCCTCCTGCATTTGATCGCACTGCCCGCAGTGCCCAATTG

GAAGCAGCCCTAGCCTATGCATCACAACATGGACCTTTAGTGTTTGACAAGGTGCGTGAT

ATTGCCATCAAAACGGCCGAAGGAGAGGGTGTGGTGCTTGTGAATACAAATTTTGATTTG

GCCCTCGCCACCTACAATGCCTGGTTCATAGGTGGTACAGCTCCAGATCCAGAGCGCCCC

ACTGAAGGTGCACCCAAATTAGTGT

>CMH-S254-15

AGATTCATGGAAAGTAGCCCATTGGTGTCTTGTGCAATAGAGTCCCTATCCTCACCAGCA

ATTGGCTATCTCAATGACATCAAGTTTGTAACCAAAGGGGGTCTCCCATCAGGAATGCCC

TTCACCTCAGTTATCAATTCGGTGAACCACATGATATACTTTGCAGCGGGTGTGCTCAAA

GCTTATGAGGACCACCACGTCCCATACACTGGCAATGTATTTCAGATAGAGACTGTCCAC

ACCTATGGTGATGATTGTATTTATAGTGTTTGTCCTGCCACCGCTTCTATCTTTGGTTCT

GTTCTCGCCAACCTGTCCTCCTTCGGTCTCAAGCCCACTGCCGCTGACAAAACTGCAGAA

ATTAAGCCCACCCAAACACCAGTTTTCCTGAAAAGAACATTCACACAGACGCCCTATGGG

GTGAGGGCACTATTGGACATCAACTCCATCATTCGGCAGTTTTACTGGGTTAAGGCGAAT

CGCACTAGTGACCCATCTAGCCCTCCTGCATTTGATCGCACTGCCCGCAGTGCCCAATTG

GAAGCAGCCCTAGCCTATGCATCACAACATGGACCTTTAGTGTTTGACAAGGTGCGTGAT

ATTGCCATCAAAACGGCCGAAGGAGAGGGTGTGGTGCTTGTGAATACAAATTTTGATTTG

GCCCTCGCCACCTACAATGCCTGGTTCATAGGTGGTACAGCTCCAGATCCAGAGCGCCCC

ACTGAAGGTGCACCCAAAATAGTGT

>CMH-S108-16

AGATTCATGGAAAGTAGCCCATTGGTGTCTTGTGCAATAGAGTCCCTGTCCTCACCAGCA

ATTGGCTATCTCAATGACATCAAGTTTGTAACCAAAGGGGGTCTCCCATCAGGAATGCCC

TTCACCTCAGTCATCAACTCGGTGAACCACATGATATACTTTGCAGCGGGTGTGCTCAAA

GCTTATGAGGACCACCACGTCCCATACACTGGCAATGTATTTCAGATAGAGACTGTCCAC

ACCTATGGTGATGATTGTATTTATAGTGTTTGTCCTGCCACCGCTTCTATCTTTGGTTCT

GTTCTCGCCAACCTGTCCTCCTTCGGTCTCAAGCCCACTGCCGCTGACAAAACTGCAGAA

ATTAAGCCCACCCAAACACCAGTTTTCCTGAAAAGAACATTCACACAGACGCCCTATGGG

GTGAGGGCACTATTGGACATCAACTCCATCATTCGGCAGTTTTACTGGGTTAAGGCGAAT

CGCACTAGTGACCCATCTAGCCCTCCTGCATTTGATCGCACTGCCCGCAGTGCCCAATTG

GAAGCAGCCCTAGCCTATGCATCACAACATGGACCTTTAGTGTTTGACAAGGTGCGTGAT

ATTGCCATCAAAACGGCCGAAGGAGAGGGTGTGGTGCTTGTGAATACAAATTCTGATTTG

GCCCTCGCCACCTACAATGCCTGGTTCATAGGTGGTACAGCTCCAGATCCAGAGCGCCCC

ACTGAAGGTGCACCCAAATTAGTGT

>CMH-ST004-16

AGATTCATGGAAAGTAGCCCATTGGTGTCTTGTGCAATAGAGTCCCTGTCCTCACCAGCA

ATTGGCTATCTCAATGACATCAAGTTTGTAACCAAAGGGGGTCTCCCATCAGGAATGCCC

TTCACCTCAGTTATCAACTCGGTGAACCACATGATATACTTTGCAGCGGGTGTGCTCAAA

GCTTATGAGGACCACCACGTCCCATACACTGGCAATGTATTTCAGATAGAGACTGTCCAC

ACCTATGGTGATGATTGTATTTATAGTGTTTGTCCTGCCACCGCTTCTATCTTTGGCTCT

GTTCTCGCCAACCTGTCCTCCTTCGGTCTCAAGCCCACTGCCGCTGACAAAACTGCAGAA

ATTAAGCCCACCCAAACACCAGTTTTCCTGAAAAGAACATTCACACAGACGCCCTATGGG

GTGAGGGCACTATTGGACATTAACTCCATCATTCGGCAGTTTTACTGGGTTAAGGCGAAT

CGCACTAGTGACCCATCTAGCCCTCCTGCATTTGATCGCACTGCCCGCAGTGCCCAATTG

GAAGCAGCCCTAGCCTATGCATCACAACATGGACCTTTAGTGTTTGACAAGGTGCGTGAT

ATTGCCATCAAAACGGCCGAAGGAGAGGGTGTGGTGCTTGTGAATACAAATTTTGATTTG

GCCCTCGCCACCTACAATGCCTGGTTCATAGGTGGTACAGCTCCAGATCCAGAGCGCCCC

ACTGAAGGTGCACCCAAATTAGTGT

>CMH-ST029-16

AGATTCATGGAAAGCAGCCCATTGGTGTCCTGTGCAATAGAGTCCCTGTCCTCACCAGCA

ATTGGCTATCTTAATGACATTAAGTTCGTAACCAAAGGGGGTCTCCCATCAGGAATGCCC

TTCACCTCAGTCATCAACTCGGTGAACCACATGATATACTTTGCAGCGGGTGTGCTCAAA

GCTTATGAGGACCACCACGTCCCATACACTGGCAATGTATTTCAGATAGAGACTGTCCAT

ACCTATGGTGATGACTGTATTTATAGTGTTTGTCCTGCCACCGCTTCTATCTTTGGTTCT

GTTCTCGCTAACCTGTCTTCCTTTGGTCTCAAGCCCACTGCCGCTGACAAAACTGCAGAA

ATCAAGCCCACCCAAACACCAGTTTTCCTGAAAAGAACATTCACACAGACGCCCTATGGG

GTGAGGGCACTGTTGGACATTAATTCCATCATTCGGCAGTTCTACTGGGTTAAGGCGAAC

CGCACCAGTGACCCATCTAGTCCCCCTGCATTTGATCGCACTGCCCGCAGTGCCCAGTTG

GAAGCAGCCCTAGCCTATGCATCACAACATGGACCTTTAGTGTTTGACAAGGTGCGTGAT

ATTGCCATCAAAACGGCCGAAGGAGAGGGTGTGGTGCTTGTGAATACAAACTTTGATTTG

GCCCTCGCCACCTACAATGCCTGGTTCATAGGTGGTACAGCTCCAGATCCAGAGCGCCCC

ACTGAAGGTGCACCCAAATTAGTGT

>CMH-R076-18

AGATTCATGGAAAGCAGCCCATTGGTGTCCTGTGCAATAGAGTCCCTGTCCTCACCAGCA

ATTGGCTATCTTAATGACATTAAATTCGTAACCAAAGGGGGTCTCCCATCAGGAATGCCC

TTCACCTCAGTCATCAACTCGGTGAACCACATGATATACTTTGCAGCGGGTGTGCTCAAA

GCTTATGAGGACCACCACGTCCCATACACTGGCAATGTATTCCAGATAGAGACTGTCCAC

ACCTATGGTGATGACTGTATTTATAGTGTTTGTCCTGCCACCGCTTCTATCTTTGGTTCT

GTTCTCGCTAACCTGTCTTCCTTTGGTCTCAAGCCCACTGCCGCTGACAAAACTGCAGAA

ATCAAGCCCACCCAAACACCAGTTTTCCTGAAAAGAACATTCACACAGACGCCCTATGGG

GTGAGGGCACTGTTGGACATTAATTCCATCATTCGGCAGTTCTACTGGGTTAAGGCGAAC

CGCACCAGTGACCCATCTAGTCCCCCTGCATTTGATCGCACTGCCCGCAGTGCCCAGTTG

GAAGCAGCCCTAGCCTATGCATCACAACATGGACCTTTAGTGTTTGACAAGGTGCGTGAT

ATTGCCATCAAAACGGCCGAAGGAGAGGGTGTGGTGCTTGTGAATACAAACTTTGATTTG

GCCCTCGCCACCTACAATGCCTGGTTCATAGGTGGTACAGCTCCAGATCCAGAGCGCCCC

ACTGAAGGTGCACCCAAATTAGTGT

>CMH-N061-18

AGATTTGCAGAACCCCACCCAATTGTTTCATGCGCCATTGAGTCACTGTCATCACCAGCA

GTTGGTTACATCAATGACATTAAGTTCACAACCAGGGGGGGCTTACCTTCGGGAATGCCA

TTCACGTCAGTTATCAACTCACTCAACCACATGATATACGTTGCTGCAGCGGTCCTGCAG

GCGTATGAAGAACACCACGCTCCCTACACTGGCAATGTTTTCCAAATTGAAACTATTCAT

ACATATGGTGATGACTGTATGTACAGTTTCTGCCCTGCCACGGCATCAGTATTCCAAACA

GTTTTGGCCAGCTTGATTCGGTTCGGATTAAAGCCCACTGCGGCGGACAAAAGTGAAGCA

ATTAAACCCACCACCACACCAGTGTTTTTGAAAAGAACGTTTACACAGACACCACAAGGG

GTTAGGGCATTGTTAGATTTGTCTTCAATCACAAGGCAATTCTACTGGTTAAAAGCCAAC

AGGACCTCAGACCCAACCAGCCCACCAGCATTTGACCGCCAAGCACGGGGTGCCCAACTG

GAAAACGCGCTGGCATTTGCCTCCCAACACGGACCGCTAGCTTTTGACAAGGTCCGTGAG

ATAGCCATCAAAACAGCACAGGGTGAGGGTTTGGTGTTAGTGAACACCAATTATGATCAT

GCTCTCGCCACCTACAACGCATGGTTCATAGGTGGTACAGTGCCTGACCCAGAACGCCCC

ACTGAAGGCGCGTCCAAAATAGTGT

>CMH-N145-12

CGGCTGTCTGAAGCAACACCAATAACAACATCGGCCGTAGAGTTACTATCATCACCAGCA

CGTGGCCACTTGAATGACATAATCTTTGTGACAAAGTCTGGACTCCCCTCGGGCATGCCA

TTCACCAGCGTTGTCAATTCACTTAACCACATGACATACTTTGCGGCCGCAGTGCTGAAG

GCCTATGAACAACATGGGGCACCTTACACTGGCAATGTGTTTCAGGTGGAAACTGTTCAC

ACATATGGTGATGATTGTATTTACTCCTTGTGCCCGGCAACTGCTTCCATTTTTGAAACT

GTTTTGGCCAACCTCAGTGCCTTTGGTCTACGGCCCACCGCTGCCGACAAGACAGACAAA

ATAGCCCCCACCCATACGCCAGTCTTTCTAAAGCGAACTCTTACCTGTACACCAAGAGGT

ATCCGTGGGCTCCTCGACATCACGTCAATTAAGCGGCAATTCTTCTGGATTAAGGCCAAC

CGCACCACAGATATATCATCCCCGCCCGCCTACGACAGGGAGGCCCGGAGTGTGCAGCTT

GAAAATGCCCTAGCCTACGCTTCACAACATGGCCATGCAATCTTTGAGGAGATTGCTGAA

ATTGCCAAGAGAACGGCACAGTCAGAAGGGTTAGTGCTCACCAATGTGAACTATGACCAG

GCTCTCGCCACCTACGAAGCGTGGTTTATAGGTGGTACAGGCACCGGCCAAGATAGCCCC

AGTGAAGAGACTACCAAATTAGTGT

>CMH-N021-13

CGGCTGTCTGAAGCAACACCAATAACAACATCGGCCGTAGAGTTACTATCGTCACCAGCA

CGTGGCCACTTGAATGACATAATCTTTGTGACAAAGTCTGGACTCCCCTCAGGCATGCCA

TTCACCAGCGTTGTCAATTCTCTCAACCACATGACATACTTTGCAGCTGCAGTGCTGAAG

GCCTATGAACAACATGGGGCACCTTACACTGGTAATGTGTTTCAGGTAGAAACTGTGCAC

ACATATGGTGATGATTGCATTTACTCCTTGTGCCCGGCGACTGCTTCTATCTTTGAAACT

GTTTTGGCCAACCTCAGTGCCTTTGGTCTACGGCCCACCGCTGCCGACAAGACAGACAAA

ATAGTCCCCACCCATACACCAGTCTTCCTAAAGCGAACTCTCACCTGTACACCAAGAGGC

ATCCGTGGGCTCCTCGACATCACGTCAATTAAGCGGCAATTCTTCTGGATCAAGGCCAAC

CGCACCACAGATATATCATCCCCGCCTGCCTACGACAGGGAGGCCCGGAGTGTGCAGCTT

GAAAATGCCCTAGCCTACGCTTCACAACATGGCCACGCAATCTTTGAGGAGATTGCTGAA

ATTGCCAAGAGAACGGCACAGTCAGAAGGGTTAGTGCTCACCAATGTGAACTATGACCAG

GCTCTCGCCACCTACGAAGCGTGGTTTATAGGTGGTACAGGCACCGGCCAAGATAGCCCC

AGTGAAGAGGCTACCAAATTAGTGT

>CMH-S034-13

CGGCTGTCTGAAGCAACACCAATAACAACATCGGCCGTAGAGTTGCTCTCATCACCAGCA

CGTGGCCATTTGAACGATATCATCTTTGTGACAAAGTCTGGACTCCCCTCAGGTATGCCA

TTCACCAGTGTTGTCAACTCTCTTAACCATATGACATACTTTGCTGCTGCAGTATTGAAG

GCCTATGAACAACATGGGGCGCCTTACACTGGCAATGTGTTTCAGGTGGAAACTGTTCAC

ACATATGGTGATGATTGCATTTACTCCCTGTGCCCAGCAACCGCTTCCATCTTTGAAACT

GTTTTGGCCAACCTCAGTGCCTTTGGTCTACGGCCCACCGCTGCCGATAAAACAGACAAA

ATAGCCCCCACCCACACACCAGTCTTTTTAAAGCGGACTCTTACCTGCACACCAAGAGGC

ATCCGTGGGCTACTTGACATCACGTCAATCAGGCGGCAATTCTTCTGGATCAAGGCAAAC

CGCACCACAGACATATTATCCCCGCCCGCTTATGACAGGGAAGCCCGAAGTGTGCAGCTT

GAAAATGCCCTAGCTTACGCCTCACAACATGGCCACGCAATTTTTGAAGAGATTGCCGAA

ATTGCCAAGAGGACGGCACAGTCAGAAGGGTTGGTGCTCACCAATGTGAACTATGACCAG

GCTCTCGCCACCTACGAAGCATGGTTTATAGGTGGTACAGGCACCGGTCAAGATAGCCCC

AGTGAAGAGACTACCAAATTAGTGT

>CMH-S050-17

CGGCTGTCTGAAGCAACACCAATAACAACGTCGGCCGTAGAGTTGCTATCATCGCCAGCA

CGTGGCCACTTGAATGACATAATTTTTGTGACAAAGTCTGGACTCCCCTCGGGCATGCCA

TTCACCAGCGTTGTTAATTCTCTTAACCACATGACATACTTTGCAGCTGCAGTGTTGAAG

GCCTATGAACAACATGGGGCACCTTACACTGGTAATGTTTTTCAGGTAGAAACTGTCCAC

ACATATGGTGATGATTGCATTTACTCCTTGTGCCCGGCGACTGCTTCCATCTTCGAAACT

GTTTTGGCCAACCTCAGTGCCTTTGGTCTACGGCCCACCGCTGCCGACAAAACAGACAAA

ATAGTCCCCACCCACACACCAGTGTTCCTAAAGCGAACTCTTACTTGTACACCAAGAGGC

ATCCGTGGGCTCCTCGACATCACGTCAATTAAGCGGCAATTCTTCTGGATCAAGGCCAAC

CGCACCACAGATATATTATCCCCGCCTGCCTACGACAGGGAGGCCCGGAGTGTGCAGCTT

GAAAACGCCCTAGCCTACGCTTCACAACATGGCCATGCAATCTTTGAGGAGATCGCTGAA

ATTGCCAAGAGAACGGCACAGTCAGAAGGGTTAGTGCTCACCAATGTGAATTATGACCAG

GCTCTCGCCACCTACGAAGCATGGTTTATAGGTGGTACAGGCACTGGCCAAGATAGCCCC

AGTGAAGAGACTACCAAATTAGTGT

>CMH-S057-17

CGGCTGTCTGAAGCAACACCAATAACAACGTCGGCCGTAGAGTTGCTATCATCGCCAGCA

CGTGGCCACTTGAATGACATAATTTTTGTGACAAAGTCTGGACTCCCCTCGGGCATGCCA

TTCACCAGCGTTGTTAATTCTCTTAACCACATGACATACTTTGCAGCTGCAGTGTTGAAG

GCCTATGAACAACATGGGGCACCTTACACTGGTAATGTTTTTCAGGTAGAAACTGTCCAC

ACATATGGTGATGATTGCATTTACTCCTTGTGCCCGGCGACTGCTTCCATCTTCGAAACT

GTTTTGGCCAACCTCAGTGCCTTTGGTCTACGGCCCACCGCTGCCGACAAAACAGACAAA

ATAGTCCCCACCCACACACCAGTGTTCCTAAAGCGAACTCTTACTTGTACACCAAGAGGC

ATCCGTGGGCTCCTCGACATCACGTCAATCAAGCGGCAATTCTTCTGGATCAAGGCCAAC

CGCACCACAGATATATTATCCCCGCCTGCCTACGACAGGGAGGCCCGGAGTGTGCAGCTT

GAAAACGCCCTAGCCTACGCTTCACAACATGGCCATGCAATCTTTGAGGAGATCGCTGAA

ATTGCCAAGAGAACGGCACAGTCAGAAGGGTTAGTGCTCACCAATGTGAATTATGACCAG

GCTCTCGCCACCTACGAAGCATGGTTTATAGGTGGTACAGGCACTGGCCAAGATAGCCCC

AGTGAAGAGACTACCAAATTAGTGT

>CMH-S120-17

CGGCTGTCTGAAGCAACACCAATAACAACATCGGCCGTAGAGTTGCTCTCATCACCAGCA

CGTGGTCATTTGAACGATATCATCTTTGTGACAAAGTCTGGACTCCCCTCAGGTATGCCA

TTCACCAGTGTTGTCAACTCTCTTAACCATATGACATACTTTGCTGCTGCAGTATTGAAG

GCCTATGAACAACATGGGGCGCCTTACACTGGTAATGTGTTTCAAGTGGAAACTGTTCAC

ACATATGGTGATGATTGCATTTACTCCCTGTGCCCAGCAACCGCTTCCATCTTTGAAACT

GTTTTGGCCAACCTCAGTGCCTTTGGTCTACGGCCCACTGCTGCTGATAAAACAGACAAA

ATAGCCCCCACCCACACACCAGTCTTTTTAAAGCGGACTCTCACCTGCACACCAAGAGGC

ATCCGTGGGCTACTCGACATCACGTCAATCAGGCGGCAATTCTTCTGGATCAAGGCCAAC

CGCACCACAGACATAACATCCCCACCCGCTTATGACAGGGAAGCCCGTAGCGTGCAGCTT

GAAAATGCCCTGGCTTATGCCTCACAACATGGCCACGCAATTTTTGAGGAGATTGCCGAA

ATTGCCAAGAGGACGGCGCAGTCAGAAGGGTTAGTGCTCACCAATGTGAACTATGACCAG

GCTCTCGCCACCTACGAAGCATGGTTCATAGGTGGTACAGGCACCGGTCAAGATAGCCCC

AGTGAAGAGACTACCAAGTTAGTGT

>CMH-ST028-17

CGGCTGTCTGAAGCAACACCAATAACAACATCGGCCGTGGAGTTGCTCTCATCACCAGCA

CGTGGCCATTTAAACGACATCATCTTTGTGACAAAGTCTGGACTCCCCTCAGGTATGCCA

TTCACCAGCGTTGTCAACTCTCTTAACCATATGACATACTTTGCTGCTGCAGTATTGAAG

GCCTATGAACAACATGGGGCGCCTTACACTGGTAATGTGTTTCAGGTGGAAACTGTTCAC

ACATATGGTGATGATTGCATCTACTCCCTGTGTCCAGCAACCGCTTCCATCTTTGAAACT

GTTTTGGCCAACCTCAGTGCCTTTGGTCTGCGGCCCACCGCTGCCGACAAAACAGATAAA

ATAGCCCCCACCCACACACCAGTCTTTTTAAAGCGGACTCTCACCTGCACACCAAGAGGC

ATCCGTGGGCTACTCGACATCACGTCAATCAGGCGGCAATTCTTCTGGATCAAGGCCAAC

CGCACCACAGACATATCATCCCCGCCCGCTTATGACAGGGAAGCCCGGAGTGTGCAGCTT

GAAAATGCACTGGCTTATGCCTCACAACATGGCCACGCAATTTTTGAGGAGATTGCCGAA

ATTGCCAAGAGAACGGCACAGTCAGAAGGGTTAGTGCTCACCAATGTGAACTATGACCAG

GCTCTCGCCACCTACGAAGCATGGTTTATAGGTGGTACAGGCACCGGTCAAGATAGCCCC

AGTGAAGAGACTACCAAGTTAGTGT

>CMH-N091-18

CGGCTGTCTGAAGCAACACCAATAACAACATCGGCCGTGGAGTTGCTCTCATCACCAGCA

CGTGGCCATTTAAACGACATCATCTTTGTGACAAAGTCTGGACTCCCCTCAGGTATGCCA

TTCACCAGCGTTGTCAACTCTCTTAACCATATGACATACTTTGCTGCTGCAGTATTGAAG

GCCTATGAACAACATGGGGCGCCTTACACTGGTAATGTGTTTCAGGTGGAAACTGTTCAC

ACATATGGTGATGATTGCATCTACTCCCTGTGTCCAGCAACCGCTTCCATCTTTGAAACT

GTTTTGGCCAACCTCAGTGCCTTTGGTCTGCGGCCCACCGCTGCCGACAAAACAGATAAA

ATAGCCCCCACCCACACACCAGTCTTTTTAAAGCGGACTCTCACCTGCACACCAAGAGGC

ATCCGTGGGCTACTCGACATCACGTCAATCAGGCGGCAATTCTTCTGGATCAAGGCCAAC

CGCACCACAGACATATCATCCCCGCCCGCTTATGACAGGGAAGCCCGGAGTGTGCAGCTT

GAAAATGCACTGGCTTATGCCTCACAACATGGCCACGCAATTTTTGAGGAGATTGCCGAA

ATTGCCAAGAGAACGGCACAGTCAGAAGGGTTAGTGCTCACCAATGTGAACTATGACCAG

GCTCTCGCCACCTACGAAGCATGGTTTATAGGTGGTACAGGCGCCGGTCAAGATAGCCCC

AGTGAAGAGACTACCAAGTTAGTGT

>CMH-N104-18

CGGCTGTCTGAAGCAACACCAATAACTACATCGGCCGTAGAGTTGCTCTCATCACCAGCA

CGTGGCCATTTGAATGATATCATCTTTGTGACAAAGTCTGGACTCCCCTCAGGTATGCCA

TTCACCAGTGTTGTCAACTCACTAAACCATATGACATACTTTGCTGCTGCAGTATTGAAG

GCCTATGAACAACATGGGGCGCCTTACACTGGTAATGTGTTTCAGGTGGAAACTGTTCAC

ACATATGGTGATGATTGCATTTACTCCCTGTGCCCAGCAACCGCTTCCATCTTTGAAACT

GTTTTGGCCAACCTCAGTGCCTTTGGTCTACGGCCCACCGCTGCCGACAAAACAGACAAA

ATAGCCCCCACCCACACACCAGTCTTTTTAAAGCGGACTCTCACCTGCACACCAAGAGGC

ATCCGTGGGTTGCTCGACATCACGTCAATCAGGCGGCAATTCTTCTGGATTAAGGCCAAC

CGCACCACAGACATAACATCCCCGCCCGCTTATGACAGAGAAGCCCGGAGTGTGCAGCTT

GAAAATGCACTGGCTTATGCTTCACAACATGGCCACGCGATTTTTGAGGAGATTGCCGAA

ATTGCCAAGAGAACAGCACAGTCAGAAGGGTTAGTGCTCACCAATGTGAACTATGACCAG

GCTCTCGCCACCTACGAAGCATGGTTTATAGGTGGTACAGGCACCGGTCAAGATAGCCCC

AGTGAAGAGACTACCAAGTTAGTGT

>CMH-R140-18

CGGCTGTCTGAAGCAACACCAATAACTACATCGGCCGTAGAGTTGCTCTCATCACCAGCA

CGTGGCCATTTGAATGATATCATCTTTGTGACAAAGTCTGGACTCCCCTCAGGTATGCCA

TTCACCAGTGTTGTCAACTCTCTAAACCATATGACATACTTTGCTGCTGCAGTATTGAAG

GCCTATGAACAACATGGGGCGCCTTACACTGGCAATGTGTTTCAGGTGGAAACTGTTCAC

ACATATGGTGATGATTGCATTTACTCCCTGTGCCCAGCAACCGCTTCCATCTTTGAAACT

GTTTTGGCCAACCTCAGTGCCTTTGGTCTACGGCCCACCGCTGCCGACAAAACAGACAAA

ATAGCCCCCACCCACACACCAGTCTTTTTAAAGCGGACTCTCACCTGCACACCAAGAGGC

ATCCGTGGGTTGCTCGACATCACGTCAATCAGGCGGCAATTCTTCTGGATTAAGGCCAAC

CGCACCACAGACATAACATCCCCGCCCGCTTATGACAGAGAAGCCCGGAGTGTGCAGCTT

GAAAATGCACTGGCTTATGCCTCACAACATGGCCACGCAATTTTTGAGGAGATTGCCGAA

ATTGCCAAGAGAACAGCACAGTCAGAAGGGTTAGTGCTCACCAATGTGAACTATGACCAG

GCTCTCGCCACCTACGAAGCATGGTTTATAGGTGGTACAGGCACCGGTCAAGATAGCCCC

AGTGAAGAGACTACCAAGTTAGTGT

>CMH-S175-18

CGGCTGTCTGAAGCAACACCAATAACTACATCGGCCGTAGAGTTGCTCTCATCGCCAGCA

CGTGGCCATTTGAATGATATCATCTTTGTGACAAAGTCTGGACTCCCCTCAGGTATGCCA

TTCACCAGTGTTGTCAACTCTCTAAACCATATGACATACTTTGCTGCTGCAGTATTGAAG

GCCTATGAACAACATGGGGCGCCTTACACTGGCAATGTGTTTCAGGTGGAAACTGTTCAC

ACATATGGTGATGATTGCATTTACTCCCTGTGCCCAGCAACCGCTTCCATCTTTGAAACT

GTTTTGGCCAACCTCAGTGCCTTTGGTCTACGGCCCACCGCTGCCGACAAAACAGACAAA

ATAGCCCCCACCCACACACCAGTCTTTTTAAAGCGGACTCTCACCTGCACACCAAGAGGC

ATCCGTGGGTTGCTCGACATCACGTCAATCAGGCGGCAATTCTTCTGGATTAAGGCCAAC

CGCACCACAGACATAACATCCCCGCCCGCTTATGATAGAGAAGCCCGGAGTGTGCAGCTT

GAAAATGCACTGGCTTATGCTTCACAACATGGCCACGCAATTTTTGAGGAGATTGCTGAA

ATTGCCAAGAGAACAGCACAGTCAGAAGGGTTAGTGCTCACCAATGTGAACTATGACCAG

GCTCTCGCCACCTACGAAGCATGGTTTATAGGTGGTACAGGCACCGGTCAAGATAGCCCC

AGTGAAGAGACTACCAAGTTAGTGT

>CMH-ST189-18

CGGCTGTCTGAAGCAACACCAATAACAACATCGGCCGTGGAGTTGCTCTCATCACCAGCG

CGTGGCCATTTGAACGATATCATTTTTGTGACAAAGTCTGGACTCCCCTCAGGTATGCCA

TTCACCAGTGTTGTCAACTCTCTTAACCATATGACATACTTTGCTGCTGCAGTATTGAAG

GCCTATGAACAACATGGGGCGCCTTACACTGGTAATGTGTTCCAGGTGGAAACTGTTCAC

ACATATGGTGATGATTGCATTTACTCCCTGTGCCCAGCAACCGCTTCCATCTTTGAAACT

GTTTTGGCCAACCTCAGTGCCTTTGGTCTGCGGCCCACCGCTGCTGATAAAACAGATAAA

ATAGCCCCCACCCACACACCAGTCTTTTTGAAGCGGACTCTTACTTGCACACCAAGAGGC

ATCCGTGGGCTACTTGACATCACGTCAATCAGGCGGCAATTTTTCTGGATCAAGGCAAAC

CGCACCACAGACATATCATCCCCGCCCGCCTATGACAGGGAAGCCCGCAGTGTGCAGCTT

GAAAATGCCCTAGCTTACGCCTCACAACATGGCCACGCAATCTTCGAAGAGATTGCCGAA

ATTGCCAAGAGGACGGCACAGTCAGAAGGGTTGGTGCTCACCAATGTGAACTATGACCAG

GCTCTCGCCACCTACGAAGCATGGTTTATAGGTGGTACAGGCACTGGTCAAGATAGCCCC

AGTGAAGAGACTACCAAGTTAGTGT

>CMH-ST091-16

AGGCTATCAGAGGCCACACCCATCACCACATCAGCAGTCGAGCTTCTTTCGTCGCCTGCC

CGTGGCCACCTCAATGACATAATCTTCATCACTAAATCTGGGCTGCCCTCGGGTATGCCA

TTCACCAGTGTGATCAATTCACTCAACCACATGACCTACTTTGCAGCAGCGGTGCTCAAG

GCCTATGAACAGCATGGTGCCCCCTACACTGGCAACGTGTTCCAGGTGGAGACTGTCCAC

ACTTACGGTGATGACTGTATATATTCACTGTGCCCAGCAACAGCATCCATTTTTGAAACT

GTCTTGGCCAACTTAAGTGCTTTTGGTCTAAAACCCACCGCAGCAGACAAGACAGAGAAG

ATTGCCCCCACTCACACCCCAGTTTTTCTCAAACGCACCCTGACATGCACCCCTCGTGGC

ATCCGAGGACTGCTCGATATTACATCAATTCGGCGACAGTTCTATTGGATTAAAGCCAAC

CGAACCACAGACATAACATCCCCACCTGCATATGATAGGGAAGCCAGGAGCGTGCAGCTC

GAAAATGCCCTGGCTTATGCCTCACAGCATGGACATGCGGTCTTTGAAGAAATTGCTGAG

ATCGCAAAGAAAACTGCTCAGGCTGAAGGGTTGGTATTGACAAACGTCAATTATGACCAG

GCTCTCGCCACCTACGAAGCATGGTTCATAGGTGGTGCAGGCACCAGTCCAGATGGCCCC

AGTGAAGAGACCACCAAATTAGTGT

>CMH-ST095-16

AGGCTATCAGAGGCCACACCCATCACCACATCAGCAGTCGAGCTTCTTTCGTCGCCTGCC

CGTGGCCACCTCAATGACATAATCTTCATCACTAAATCTGGGCTGCCCTCGGGTATGCCA

TTCACCAGTGTGATCAATTCACTCAACCACATGACCTACTTTGCAGCAGCGGTGCTCAAG

GCCTATGAACAGCATGGTGCCCCCTACACTGGCAACGTGTTCCAGGTGGAGACTGTCCAC

ACTTACGGTGATGACTGTATATATTCACTGTGCCCAGCAACAGCATCCATTTTTGAAACT

GTCTTGGCCAACTTAAGTGCTTTTGGTCTAAAACCCACCGCAGCAGACAAGACAGAGAAG

ATTGCCCCCACTCACACCCCAGTTTTTCTCAAACGCACCCTGACATGCACCCCTCGTGGC

ATCCGAGGACTGCTCGATATTACATCAATTCGGCGACAGTTCTATTGGATTAAAGCCAAC

CGAACCACAGACATAACATCCCCACCTGCATATGATAGGGAAGCCAGGAGCGTGCAGCTC

GAAAATGCCCTGGCTTATGCCTCACAGCATGGACATGCGGTCTTTGAAGAAATTGCTGAG

ATCGCAAAGAAAACTGCTCAGGCTGAAGGGTTGGTATTGACAAACGTCAATTATGACCAG

GCTCTCGCCACCTACGAAGCATGGTTCATAGGTGGTGCAGGCACCAGTCCAGATGGCCCC

AGTGAAGAGACCACCAAATTAGTGT

>CMH-R089-18

AGGCTTTCAGAGGCCACACCCATTACTACATCAGCAGTTGAGCTTCTCTCATCACCTGCC

CGTGGCCACCTCAATGATATAGTCTTCATCACTAAATCTGGGCTGCCCTCGGGCATGCCT

TTCACTAGTGTAATAAACTCACTCAACCACATGACCTACTTTGCAGCAGCAGTGCTCAAG

GCCTATGAACAACATGGTGCCCCTTACACCGGCAATGTGTTTCAAGTGGAAACTGTCCAC

ACCTATGGTGATGACTGCATATATTCATTGTGCCCAGCAACAGCATCTATTTTTGACACT

GTCCTGGCCAATTTGAGTGCTTTTGGTCTAAAACCTACTGCAGCAGACAAAACAGAGAAA

ATCACCCCTACCCACACCCCAGTCTTTCTCAAACGCACTCTGACATGTACTCCTCGCGGC

ATCCGAGGACTGCTTGACATCACATCAATTCGGCGACAGTTTTACTGGATTAAGGCCAAC

CGCACCACAGACATAACATCCCCACCCGCATATGACAGGGAAGCCAGAAGTGTGCAGCTT

GAAAATGCCCTGGCTTATGCCTCACAGCATGGTCATGCGGTCTTTGAAGAAATTGCCGAG

ATCGCAAAGAAAACTGCTCAGGCTGAGGGGTTGGTGTTAACAAATGTGAATTATGACCAG

GCTCTCGCCACCTACGAAGCGTGGTTCATAGGTGGTGCAGGCACCAGTCCAGATGGCCCC

AGTGAAGAGACCACCAAATTAGTGT

>CMH-ST202-18

AGGTTATCAGAGGCCACACCCATCACCACATCAGCAGTCGAGCTTCTTTCGTCGCCTGCC

CGTGGCCACCTCAATGACATAGTCTTCATCACTAAATCTGGGCTGCCCTCGGGTATGCCA

TTCACCAGTGTGATCAATTCACTCAACCACATGACCTATTTTGCAGCAGCGGTGCTCAAG

GCCTATGAACAACATGGTGCCCCCTACACTGGCAATGTGTTCCAGGTGGAGACTGTCCAC

ACTTATGGTGATGACTGTATATATTCACTGTGCCCAGCAACAGCATCCATTTTTGAAACT

GTCTTGGCCAACTTAAGTGCTTTTGGTCTAAAACCCACTGCAGCAGACAAAACAGAGAAA

ATTGCCCCCACTCACACCCCAGTTTTTCTCAAACGCACCCTGACATGCACCCCTCGTGGC

ATCCGAGGACTGCTCGACATTACATCAATTCGGCGACAGTTCTTTTGGATTAAAGCCAAC

CGAACCACAGACATAACATCCCCACCTGCATACGATAGGGAAGCCAGGAGCGTGCAGCTC

GAAAATGCCCTGGCTTATGCCTCACAGCATGGACATGCGGTCTTTGAAGAAATTGCTGAG

ATCGCAAAGAAAACTGCTCAGGCTGAAGGGTTGGTGTTGACAAATGTCAATTATGACCAG

GCTCTCGCCACCTACGAAGCATGGTTCATAGGTGGTGCAGGCACCAGTCCAGATGGCCCC

AGTGAAGAGACCACCAAATTAGTGT

>CMH-ST207-18

AGGTTATCAGAGGCCACACCCATCACCACATCAGCAGTCGAGCTTCTTTCGTCGCCTGCC

CGTGGCCACCTCAATGACATAGTCTTCATCACTAAATCTGGGCTGCCCTCGGGTATGCCA

TTCACCAGTGTGATCAATTCACTCAACCACATGACCTATTTTGCAGCAGCGGTGCTCAAG

GCCTATGAACAACATGGTGCCCCCTACACTGGCAATGTGTTCCAGGTGGAGACTGTCCAC

ACTTATGGTGATGACTGTATATATTCACTGTGCCCAGCAACAGCATCCATTTTTGAAACT

GTCTTGGCCAACTTAAGTGCTTTTGGTCTAAAACCCACTGCAGCAGACAAAACAGAGAAA

ATTGCCCCCACTCACACCCCAGTTTTTCTCAAACGCACCCTGACATGCACCCCTCGTGGC

ATCCGAGGACTGCTCGACATTACATCAATTCGGCGACAGTTCTTTTGGATTAAAGCCAAC

CGAACCACAGACATAACATCCCCACCTGCATACGATAGGGAAGCCAGGAGCGTGCAGCTC

GAAAATGCCCTGGCTTATGCCTCACAGCATGGACATGCGGTCTTTGAAGAAATTGCTGAG

ATCGCAAAGAAAACTGCTCAGGCTGAAGGGTTGGTGTTGACAAATGTCAATTATGACCAG

GCTCTCGCCACCTACGAAGCATGGTTCATAGGTGGTGCAGGCACCAGTCCAGATGGCCCC

AGTGAAGAGACCACCAAATTAGTGT

>CMH-ST247-18

AGGCTTTCAGAGGCCACACCCATTACCACATCAGCAGTTGAGCTTCTCTCATCACCTGCC

CGTGGCCACCTCAATGATATAGTCTTCATCACTAAATCTGGGCTGCCCTCGGGCATGCCT

TTCACTAGTGTGATAAACTCACTCAACCACATGACCTACTTTGCAGCAGCAGTGCTCAAG

GCCTATGAACAACATGGTGCCCCTTACACCGGCAACGTATTTCAAGTGGAAACTGTCCAC

ACCTATGGTGATGACTGCATATATTCATTGTGCCCAGCAACAGCATCTATTTTTGACACT

GTCCTGGCCAATTTGAGTGCTTTTGGTCTAAAACCCACTGCAGCAGACAAAACTGAGAAA

ATCACCCCTACCCACACCCCAGTCTTTCTCAAACGCACTCTGACATGTACTCCTCGCGGC

ATCCGAGGACTGCTTGACATCACATCAATTCGGCGACAGTTTTACTGGATTAAGGCCAAC

CGCACCACAGACATAACATCCCCCCCCGCATATGACAGGGAAGCCAGAAGTGTGCAGCTC

GAAAATGCCCTGGCTTATGCCTCACAGCATGGTCATGCGGTCTTTGAAGAAATTGCCGAG

ATCGCAAAGAAAACTGCTCAGGCTGAGGGGTTGGTGTTAACAAATGTGAATTATGACCAG

GCTCTCGCCACCTACGAAGCGTGGTTCATAGGTGGTGCAGGCACCAGTCCAGATGGCCCC

AGTGAAGAGACCACCAAATTAGTGT

>CMH-ST270-18

AGGCTTTCAGAGGCCACACCCATTACCACATCAGCAGTTGAGCTTCTCTCATCACCTGCC

CGTGGCCACCTCAATGATATAGTCTTCATCACTAAATCTGGGCTGCCCTCGGGCATGCCT

TTCACTAGTGTGATAAACTCACTCAACCACATGACCTACTTTGCAGCAGCAGTGCTCAAG

GCCTATGAACAACATGGTGCCCCTTACACCGGCAACGTATTTCAAGTGGAAACTGTCCAC

ACCTATGGTGATGACTGCATATATTCATTGTGCCCAGCAACAGCATCTATTTTTGACACT

GTCCTGGCCAATTTGAGTGCTTTTGGTCTAAAACCCACTGCAGCAGACAAAACTGAGAAA

ATCACCCCTACCCACACCCCAGTCTTTCTCAAACGCACTCTGACATGTACTCCTCGCGGC

ATCCGAGGACTGCTTGACATCACATCAATTCGGCGACAGTTTTACTGGATTAAGGCCAAC

CGCACCACAGACATAACATCCCCCCCCGCATATGACAGGGAAGCCAGAAGTGTGCAGCTC

GAAAATGCCCTGGCTTATGCCTCACAGCATGGTCATGCGGTCTTTGAAGAAATTGCCGAG

ATCGCAAAGAAAACTGCTCAGGCTGAGGGGTTGGTGTTAACAAATGTGAATTATGACCAG

GCTCTCGCCACCTACGAAGCGTGGTTCATAGGTGGTGCAGGCACCAGTCCAGATGGCCCC

AGTGAAGAGACCACCAAATTAGTGT

>CMH-N061-12

CGGTTATCTGAAGCCACCCCGATCACCACATCCGCAGTGGAGTTACTATCATCCCCGGCG

CGAGGCCACTTAAACGACATAATTTTTGTCACCAAGTCCGGGCTTCCATCAGGCATGCCA

TTCACCAGTGTCATCAATTCCCTCAACCACATGACATATTTCGCCGCGGCAGTGTTGCGG

GCCTACGAGCAACACGGGGCCCCTTACACCGGAAATGTTTTCCAAGTTGAGACAGTACAC

ACTTATGGAGATGATTGTCTGTATTCAGTCTGCCCTGCCACGGCCTCCATCTTCCACTCT

GTTTTGGCCAATCTCACCTCGTTTGGACTAAAGCCTACTGCTGCAGACAAGAGTGAATGC

ATTGCCCCCACCCACACGCCAGTATTTCTTAAAAGAACCCTGACCTGTACGCCTCGTGGC

GTGCGGGGGCTCTTGGACATAACCTCCATCAAAAGGCAGTTCCTTTGGATCAAGGCCAAT

AGAACAACTGACATCAATTCCCCACCGGCATATGACCGGGAGGCGCGTAGTGTCCAGCTC

GAAAACGCCCTTGCCTATGCTTCACAGCACGGCCACAGTGTCTTTGAGGAGATTGCCAAG

CTGGCCCGCCACACAGCCAAAGCTGAGGGGCTTGTGTTGACGAATGTCAACTACGACCAG

GCTCTCGCCACCTACGAATCCTGGTTCATAGGTGGTACAGGTCTGGTGCGAGATGGCCCC

AGTGAAGAGACCACCAAATTAGTGT

>CMH-N028-18

CGATTATCTGAAGCCACCCCAATCACCACATCCGCAGTGGAGTTGCTATCATCCCCGGCG

CGAGGCCACTTAAACGACATAATTTTTGTCACCAAGTCCGGGCTTCCATCAGGCATGCCA

TTTACCAGTGTCATCAATTCCCTCAACCACATGACATATTTTGCTGCGGCAGTGCTGCGG

GCCTACGAGCAACACGGGGCCCCTTACACCGGAAATGTTTTCCAAGTTGAGACAGTACAC

ACTTATGGAGATGACTGTCTGTATTCAGTCTGCCCTGCCACGGCCTCCATCTTCCACTCT

GTTTTGGCCAATCTCACCTCATTTGGACTAAAGCCTACTGCTGCAGACAAGAGTGAATGC

ATTGCCCCCACTCACACGCCAGTGTTTCTCAAAAGAACCCTGACCTGTACGCCTCGTGGC

GTGCGGGGGCTCTTGGACATAACCTCCATCAAAAGGCAGTTCCTTTGGATCAAGGCCAAT

AGAACAACTGACATCAATTCCCCACCAGCATATGACCGGGAGGCGCGTAGTGTCCAGCTC

GAAAACGCCCTTGCCTATGCTTCACAACACGGTCACAGTGTCTTTGAGGAGGTTGCCAAG

CTGGCCCGTCACACGGCCAAAGCTGAGGGGCTTGTGCTGACGAATGTCAACTACGACCAG

GCTCTCGCCACCTACGAATCCTGGTTCATAGGTGGTACAGGCCTGGTGCAAGATGGCCCC

AGTGAAGAGACCACCAAATTAGTGT
