# Supplementary material for: Genetic recombination and diversity of sapovirus in pediatric patients with acute gastroenteritis in Thailand, 2010–2018
Source: PeerJ. 2020 Feb 6;8:e8520. doi: 10.7717/peerj.8520 (PMC7007980; doi:10.7717/peerj.8520)
Supplement: Supplemental Information 3 [file peerj-08-8520-s003.docx]

VP1 sequences

>CMH-N018-10

ACCCACAAAATAGTGTTTGAGATGGAGGGCAATGGCTCCAACCCAGAGCCAAAACAGAGC

AATAACCCAATGGTCGTTGACCCGCCTGGCACAACAGGTCCGACCACATCCCACGTTGTT

GTTGCTAATCCGGAGCAACCCAATGGGGCCGCACAGCGCCTGGAGTTGGCTGTTGCCACT

GGTGCAATCCAATCCAATGTCCCTGAGGCAATACGCAACTGCTTTGCAGTCTTTCGTACT

TTTGCTTGGAACGACAGGATGCCCACGGGAACTTTTCTTGGATCTATATCGCTTCATCCC

AACATTAA

>CMH-S050-10

ACCCACAAAATAGTGTTTGAGATGGAGGGCAATGGCTCCAACTCAGAGCCAAAGCAGAGC

AACAACCCAATGGTCGTTGACCCGCCTGGCACAACAGGTCCGACCACATCCCATGTTGTT

GTTGCTAATCCGGAGCAACCCAATGGGGCCGCACAGCGCCTGGAGTTGGCTGTTGCCACT

GGTGCAATCCAATCCAATGTCCCTGAGGCAATACGCAACTGCTTTGCAGTCTTTCGTACT

TTTGCTTGGAACGACAGGATGCCCACGGGAACTTTTCTTGGATCTATATCGCTTCATCCC

AACATTAA

>CMH-N131-13

ACCCACAAAATAGTGTTTGAGATGGAGGGCAATGGCTCCAACTCAGAGCCAAAGCAGAGC

AACAACCCAATGGTCGTTGACCCGCCTGGCACAACAGGTCCGACCACATCCCACGTTGTT

GTTGCTAATCCGGAGCAACCCAATGGGGCCGCACAGCGCTTGGAGTTGGCTGTTGCCACT

GGTGCAATCCAATCCAATGTCCCTGAGGCAATACGCAACTGCTTTGCAGTCTTTCGTACT

TTTGCTTGGAACGACAGGATGCCCACGGGAACTTTTCTTGGATCTATATCGCTTCATCCC

AACATTAA

>CMH-S004-13

ACCCACAAAATAGTGTTTGAGATGGAGGGCAATGGCTCCAACTCAGAGCCAAAGCAGAGC

AACAACCCAATGGTCGTTGACCCGCCTGGCACAACAGGTCCGACCACATCCCACGTTGTT

GTTGCTAATCCGGAGCAACCCAATGGGGCCGCACAGCGCTTGGAGTTGGCTGTTGCCACT

GGTGCAATCCAATCCAATGTCCCTGAGGCAATACGCAACTGCTTTGCAGTCTTTCGTACT

TTTGCTTGGAACGACAGGATGCCCACGGGAACTTTTCTTGGATCTATATCGCTTCATCCC

AACATTAA

>CMH-N006-15

ACCcACAAAATAGTGTTTGAGATGGAGGGCAATGGCTCCAACTCAGAGCCAAAGCAGAGC

AACAACCCAATGGTCGTTGACCCGCCTGGCACAACAGGTCCGACCACATCCCACGTTGTT

GTTGCTAATCCGGAGCAACCCAATGGGGCCGCACAGCGCCTGGAGTTGGCTGTTGCCACT

GGTGCAGTCCAATCCAATGTCCCTGAGGCAATACGCAACTGCTTTGCAGTCTTTCGTACT

TTTGCTTGGAATGACAGGATGCCCACGGGAACTTTCCTTGGATCTATATCGCTTCATCCC

AACATTAA

>CMH-N007-15

ACCCACAAAATAGTGTTTGAGATGGAGGGCAATGGCTCCAACTCAGAGCCAAAGCAGAGC

AACAACCCAATGGTCGTTGACCCGCCTGGCACAACAGGTCCGACCACATCCCACGTTGTT

GTTGCTAATCCGGAGCAACCCAATGGGGCCGCACAGCGCCTGGAGTTGGCTGTTGCCACT

GGTGCAGTCCAATCCAATGTCCCTGAGGCAATACGCAACTGCTTTGCAGTCTTTCGTACT

TTTGCTTGGAATGACAGGATGCCCACGGGAACTTTCCTTGGATCTATATCGCTTCATCCC

AACATTAA

>CMH-S166-15

ACCCACAAAATAGTGTTTGAGATGGAGGGCAATGGCTCCAACTCAGAGCCAAAGCAGAGC

AACAACCCAATGGTCGTTGACCCGCCTGGCACAACAGGTCCGACCACATCCCACGTTGTT

GTTGCTAATCCGGAGCAACCCAATGGGGCCGCACAGCGCCTGGAGTTGGCTGTTGCCACT

GGTGCAGTCCAATCCAATGTCCCTGAGGCAATACGCAACTGCTTTGCAGTCTTTCGTACT

TTTGCTTGGAATGACAGGATGCCCACGGGAACTTTCCTTGGATCTATATCGCTTCATCCC

AACATTAA

>CMH-S198-16

ACCCACAAAATAGTGTTTGAGATGGAGGGCAATGGCTCCAATTCAGAGCCAAAGCAGAGC

AACAACCCGATGGTCGTTGACCCGCCTGGCACAACAGGTCCGACCACATCCCACGTTGTT

GTTGCTAATCCGGAGCAACCCAATGGGGCCGCACAGCGCCTGGAGCTGGCTGTTGCCACT

GGTGCAATCCAATCCAATGTCCCTGAGGCAATACGCAACTGCTTTGCAGTCTTTCGTACT

TTTGCTTGGAACGACAGGATGCCCACGGGAACTTTTCTTGGATCTATATCGCTACATCCC

AACATTAA

>CMH-S229-16

ACCCACAAAATAGTGTTTGAGATGGAGGGCAATGGCTCCAATTCAGAGCCAAAGCAGAGC

AACAACCCGATGGTCGTTGACCCGCCTGGCACAACAGGTCCGACCACATCCCATGTTGTT

GTTGCTAATCCGGAGCAACCCAATGGGGCCGCACAGCGCCTGGAGCTGGCTGTTGCCACT

GGTGCAATCCAATCCAATGTCCCTGAGGCAATACGCAACTGCTTTGCAGTCTTTCGTACT

TTTGCTTGGAACGACAGGATGCCCACGGGAACTTTTCTTGGATCTATATCGCTTCATCCC

AACATTAA

>CMH-ST016-16

ACCCACAAAATAGTGTTTGAGATGGAGGGCAATGGCTCCAATTCAGAGCCAAAGCAGAGC

AACAACCCGATGGTCGTTGACCCGCCTGGCACAACAGGTCCGACCACATCCCACGTTGTT

GTTGCTAATCCGGAGCAACCCAATGGGGCCGCACAGCGCCTGGAGCTGGCTGTTGCCACT

GGTGCAATCCAATCCAATGTCCCTGAGGCAATACGCAACTGCTTTGCAGTCTTTCGTACT

TTTGCTTGGAACGACAGGATGCCCACGGGAACTTTTCTTGGATCTATATCGCTTCATCCC

AACATTAA

>CMH-ST090-16

ACCCACAAAATAGTGTTTGAGATGGAGGGCAATGGCTCCAACTCAGAGCCAAAGCAAAGC

AATAACCCAATGGTCGTTGACCCGCCTGGCACAACAGGTCCGACCACATCCCACGTTGTT

GTTGCTAATCCGGAGCAACCCAATGGGGCCGCACAGCGCCTGGAGTTGGCTGTTGCCACT

GGTGCAATCCAATCCAATGTCCCTGAGGCAATACGCAACTGCTTTGCAGTCTTTCGTACT

TTTGCTTGGAACGACAGGATGCCCACGGGAACTTTTCTTGGATCTATATCGCTTCATCCC

AACATTAA

>CMH-ST163-16

ACCCACAAAATAGTGTTTGAGATGGAGGGCAATGGCTCCAATTCAGAGCCAAAGCAGAGC

AACAACCCGATGGTCGTTGACCCGCCTGGCACAACAGGTCCGACCACATCCCACGTTGTT

GTTGCTAATCCGGAGCAACCCAATGGGGCCGCACAGCGCCTGGAGCTGGCTGTTGCCACT

GGTGCAATCCAATCCAATGTCCCTGAGGCAATACGCAACTGCTTTGCAGTCTTTCGTACT

TTTGCTTGGAACGACAGGATGCCCACGGGAACTTTTCTTGGATCTATATCGCTACATCCC

AACATTAA

>CMH-ST199-16

ACCCACAAAATAGTGTTTGAGATGGAGGGCAATGGCTCCAACCCAGAGCCAAAGAACAGC

AACAACCCAATGGTCGTTGACCCGCCTGGCACAACAGGTCCGACCACATCCCACGTTGTT

GTTGCTAATCCGGAGCAACCCAATGGGGCCGCACAGCGCCTGGAGTTGGCTGTTGCCACT

GGTGCAATCCAATCCAATGTCCCTGAGGCAATACGCAACTGCTTTGCAGTCTTTCGTACT

TTTGCTTGGAACGACAGGATGCCCACGGGAACTTTTCTTGGATCTATATCGCTTCATCCC

AACATTAA

>CMH-S003-17

ACCCACAAAATAGTGTTTGAGATGGAGGGCAATGGCTCCAATTCAGAGCCAAAGCAGAGC

AACAACCCGATGGTCGTTGACCCGCCTGGCACAACAGGTCCGACCACATCCCACGTTGTT

GTTGCTAATCCGGAGCAACCCAATGGGGCCGCACAGCGCCTGGAGCTGGCTGTTGCCACT

GGTGCAATCCAATCCAATGTCCCTGAGGCAATACGCAACTGCTTTGCAGTCTTTCGCACT

TTTGCTTGGAACGACAGGATGCCCACGGGAACTTTTCTTGGATCTATATCGCTTCATCCC

AACATtAA

>CMH-S023-17

ACCCACAAAATAGTGTTTGAGATGGAGGGCAATGGCTCCAACTCAGAGCCAAAGCAGAGC

AACAACCCAATGGTCGTTGACCCGCCTGGCACAACAGGTCCGACCACATCCCACGTTGTT

GTTGCTAATCCGGAGCAACCCAATGGGGCCGCACAGCGCCTGGAGTTGGCTGTTGCCACT

GGTGCAATCCAATCCAATGTCCCTGAGGCAATACGCAACTGCTTTGCAGTCTTTCGTACT

TTTGCTTGGAACGACAGGATGCCCACGGGAACTTTTCTTGGATCTATATCGCTTCATCCC

AACATtAA

>CMH-S089-17

ACCCACAAAATAGTGTTTGAGATGGAGGGCAATGGCTCCAACCCAGAGCCAAAGCAGAGC

AACAACCCAATGGTCGTTGACCCGCCTGGCACAACAGGTCCGACCACATCCCACGTTGTT

GTTGCTAATCCGGAGCAACCCAATGGGGCCGCACAGCGCCTGGAGTTGGCTGTTGCCACT

GGTGCAATCCAATCCAATGTCCCTGAGGCAATACGCAACTGCTTTGCAGTCTTTCGTACT

TTTGCTTGGAACGACAGGATGCCCACGGGAACTTTCCTTGGATCTATATCGCTTCATCCC

AACATTAA

>CMH-R031-18

ACCCACAAAATAGTGTTTGAGATGGAGGGCAATGGCTCCAATTCAGAGCCAAAGCAGAGC

AACAACCCGATGGTCGTTGACCCGCCTGGCACAACAGGTCCGACCACATCCCACGTTGTT

GTTGCTAATCCGGAGCAACCCAATGGGGCCGCACAGCGCCTGGAGCTGGCTGTTGCCACT

GGTGCAATCCAATCCAACGTCCCTGAGGCAATACGCAACTGCTTTGCAGTCTTTCGTACT

TTTGCTTGGAACGACAGGATGCCCACGGGAACTTTTCTTGGATCTATATCGCTTCATCCC

AACATTAA

>CMH-S174-18

ACCCACAAATtAGTGTTTGAGATGGAGGGCAATGGCTCCAACTCAGAGTCAAAGCAGAGC

AACAACCCAATGGTCGTTGACCCGCCTGGCACAACAGGTCCGACCACATCCCACGTTGTT

GTTGCTAATCCGGAGCAACCCAATGGGGCCGCACAGCGCCTGGAGTTGGCTGTTGCCACT

GGTGCAATCCAATCCAATGTCCCTGAGGCAATACGCAACTGCTTTGCAGTCTTTCGCACT

TTTGCTTGGAACGACAGGATGCCCACGGGAACTTTTCTTGGATCTATATCGCTTCATCCC

AACATTAA

>CMH-ST097-18

ACCCACAAAATAGTGTTTGAGATGGAGGGCAATGGCTCCAATTCAGAGCCAAAGCAGAGC

AACAACCCGATGGTCGTTGACCCGCCTGGCACAACAGGTCCGACCACATCCCACGTTGTT

GTTGCTAATCCGGAGCAACCCAATGGGGCCGCACAGCGCCTGGAGCTGGCTGTTGCCACT

GGTGCAATCCAATCCAACGTCCCTGAGGCAATACGCAACTGCTTTGCAGTCTTTCGTACT

TTTGCTTGGAACGACAGGATGCCCACGGGAACTTTTCTTGGATCTATATCGCTTCATCCC

AACATtAA

>CMH-ST169-18

ACCCACAAAATAGTGTTTGAGATGGAGGGCAATGGCTCCAACTCAGAGTCAAAGCAGAGC

AACAACCCAATGGTCGTTGACCCGCCTGGCACAACAGGTCCGACCACATCCCACGTTGTT

GTTGCTAATCCGGAGCAACCCAATGGGGCCGCACAGCGCCTGGAGTTGGCTGTTGCCACT

GGTGCAATCCAATCCAATGTCCCTGAGGCAATACGCAACTGCTTTGCAGTCTTTCGTACT

TTTGCTTGGAACGACAGGATGCCCACGGGAACTTTTCTTGGATCTATATCGCTTCACCCC

AACATTAA

>CMH-S152-15

GCACCCAAATTAGTGTTTGAAATGGAGGGCAATGGCTCCCAGTTGCCAACCAATCAAAAT

GGTGGCCATCTTGGTCAGGATGTTGACCCGCCTGGCGCGACTGGTCCGACCACATCCCAT

GTTGTTGTGTCTAATCCAGAACAACCCAATGGGCCCGCACAACGCCTGGAAATGGCTGTT

GCTACTGGTTCCATCCAATCAAATGTCCCTGAAGCGATACGCAACTGCTTTGCAGTCTGT

CGTACTTTTGCTTGGAATGACAGAATGCCCACTGGAACTTTCCTGGGATCTTTATCGCTT

CATCCCAACATTAA

>CMH-S252-15

GCACCCAAATTAGTGTTTGAGATGGAGGGCAATGGCTCCCAATTGCCAACCAATCAAAAT

GGTGGCCATGTTGGCCAGGATGTTGACCCACCTGGCGCGACTGGTCCGACCACATCCCAT

GTTGTTGTGTCTAATCCAGAACAACCCAATGGGCCCGCACAACGCCTGGAAATGGCTGTT

GCTACTGGTTCCATCCAATCAAATGTCCCTGAAGCGATACGCAACTGCTTTGCAGTCTGT

CGTACTTTTGCTTGGAATGACAGAATGCCCACTGGAACTTTCCTGGGATCTTTATCGCTT

CATCCCAACATTAA

>CMH-S254-15

GCACCCAAATTAGTGTTTGAGATGGAGGGCAATGGCTCCCAATTGCCAACCAATCAAAAT

GGTGGCCATGTTGGCCAGGATGTTGACCCGCCTGGCGCGACTGGTCCGACCACATCCCAT

GTTGTTGTGTCTAATCCAGAACAACCCAATGGGCCCGCACAACGCCTGGAAATGGCTGTT

GCTACTGGTTCCATCCAATCAAATGTCCCTGAAGCGATACGCAACTGCTTTGCAGTCTGT

CGTACTTTTGCTTGGAATGACAGAATGCCCACTGGAACTTTCCTGGGATCTTTATCGCTT

CATCCCAACATTAA

>CMH-S108-16

GCACCCAAATTAGTGTTTGAGATGGAGGGCAATGGCTCCCAATTGCCAACCAATCAAAAT

GGTGGCCATGTTGGCCAGGATGTTGACCCGCCTGGCGCGACTGGTCCGACCACATCCCAT

GTTGTTGTGTCTAATCCAGAACAACCCAATGGGCCCGCACAACGCCTGGAAATGGCTGTT

GCTACTGGTTCCATCCAATCAAATGTCCCTGAAGCGATACGCAACTGCTTTGCAGTCTGT

CGTACTTTTGCTTGGAATGACAGAATGCCCACTGGAACTTTCCTGGGATCTTTATCGCTT

CATCCCAACATTAA

>CMH-ST004-16

GCACCCAAATTAGTGTTTGAGATGGAGGGCAATGGCTCCCAATTGCCAACCAATCAAAAT

GGTGGCCATGTTGGCCAGGATGTTGACCCACCTGGCGCGACTGGTCCGACCACATCCCAT

GTTGTTGTGTCTAATCCAGAACAACCCAATGGGCCCGCACAACGCCTGGAAATGGCTGTT

GCTACTGGTTCCATCCAATCAAATGTCCCTGAAGCGATACACAACTGCTTTGCAGTCTGT

CGTACTTTTGCTTGGAATGACAGAATGCCCACTGGAACTTTCCTGGGATCTTTATCGCTT

CATCCCAACATTAA

>CMH-ST029-16

GCACCCAAATTAGTGTTTGAGATGGAGGGCAATGGCTCCCAGTTGCCAACCAATCAAAAT

GGTGGCCATCTTGGCCAGGATGTTGACCCGCCTGGCGCGACTGGTCCGACCACATCCCAT

GTTGTTGTGTCTAATCCAGAACAACCCAATGGGCCCGCACAACGCCTGGAAATGGCTGTT

GCTACTGGTTCCATCCAATCAAATGTCCCTGAAGCGATACGCAACTGCTTTGCAGTCTGT

CGTACTTTTGCTTGGAATGACAGAATGCCCACTGGAACTTTCCTGGGATCTTTATCGCTT

CATCCCAACATTAA

>CMH-R076-18

GCACCCAAATTAGTGTTTGAGATGGAGGGCAATGGCTCCCAGTTGCCAACCAATCAAAAT

GGTGGCCATGTTGGCCAGGATGTTGACCCGCCTGGCGCGACTGGTCCGACCACATCCCAT

GTTGTTGTGTCTAATCCAGAACAACCCAATGGGCCCGCACAACGCCTGGAAATGGCTGTT

GCTACTGGTTCCATCCAATCAAATGTCCCTGAAGCGATACGCAACTGCTTTGCAGTCTGT

CGTACTTTTGCTTGGAATGACAGAATGCCCACTGGAACTTTCCTGGGATCTTTATCGCTT

CATCCCAACATTAA

>CMH-N061-18

GCGTCCAAAATAGTGTTTGAGATGGAGGGCAATGGCTCCCAACAAGGGGCACGACCAAAA

AGTCCACCTCAAAGTGTTGACCTTCCTGGCACGGTTGGCCCGACCACATCCAATGTTGTT

GTGGCTAATCCGGAACAACCCAATGGGACCGCACAACGCTTGGAGATGGCTGTTGCCACT

GGCACAATCCAATCCAATGTCCCTGAGGCAATACGGAACTGTTTTGCAGTCTTTCGTACT

TTTGCTTGGAATGACAGGATGCCCGCGGGAACTTACCTTGGATCTGTATCGCTTCATCCC

AACATTAA

>CMH-S034-13

aCTACCAAGTTAGTGTTTGAaaTGGAGGGCTTGGGCCAGCCACAGTCCCAAAGGGACCAA

CAGGTTATGGAACAGGTTGTCACCCCCCAGGACACCATTGGACCAACAAGTGCACTTCTA

TTGCCCACTCAAGTTGAGACACCAAATGCTAGTGCTCAGCGTGTGGAACTTGCAATGGCC

ACAGGGGCAGTGACCAGCAATGTGCCCAACTGCATCCGAGAGTGTTTTGCTGCGGTCACC

ACAATACCATGGACCACCCGGCAAGCAGCAAACACCTTTCTCGGTGCCATACATCTGGGA

CCACGCATTAA

>CMH-S120-17

ACTACCAAGTTAGTGTTTGAAATGGAGGGCTTGGGCCAGCCGCAGTCCCAAAGGGACCAA

CAGGTCATGGAACAGGTTGTCACCCCCCAGGACACCATTGGGCCAACGAGTGCACTTCTA

TTGCCCACTCAAGTTGAGACACCAAATGCTAGTGCTCAGCGAGTGGAACTTGCAATGGCC

ACAGGGGCAGTGACCAGCAATGTGCCCAACTGCATCCGAGAGTGTTTTGCTGCGGTCACC

ACAATACCATGGACCACCCGGCAAGCAGCAAACACCTTTCTCGGTGCCATACATCTGGGT

CCACGCATTAA

>CMH-ST028-17

ACTACCAAGTTAGTGTTTGAAATGGAGGGCTTGGGCCAGCCACAGTCCCAAAGGGATCAA

CAGGTTATGGAACAGGTCGTCACCCCCCAGGACACCATTGGACCAACGAGTGCACTTCTA

TTGCCCACTCAAGTTGAGACACCAAATGCCAGTGCACAGCGTGTGGAACTTGCAATGGCC

ACAGGGGCAGTGACCAGCAATGTGCCCAACTGCATCCGAGAGTGTTTTGCTGCGGTCACC

ACAATACCATGGACCACCCGACAAGCAGCAAACACCTTTCTCGGTGCCATACATCTCGCT

CccACGaTCAa

>CMH-N091-18

ACTACCAAGTTAGTGTTTGAAATGGAGGGCTTGGGCCAGCCACAGTCCCAAAGGGACCAA

CAGGTTATGGAACAGGTCGTCACCCCCCAGGACACCATTGGACCAACGAGTGCACTTTTA

TTGCCCACTCAAGTTGAGACACCAAATGCCAGTGCACAGCGTGTGGAACTTGCAATGGCC

ACAGGGGCAGTGACCAGCAATGTGCCCAACTGCATCCGAGAGTGTTTTGCTGCGGTCACC

ACAATACCATGGACCACCCGACAAGCAGCAAACACCTTTCTCGGTGCCATACATCTGGGC

CCACGCATTAA

>CMH-N104-18

ACTACCAAGTTAGTGTTTGAAATGGAGGGCTTGGGCCAGCCACAGTCCCAAAGGGACCAA

CAGGTTATGGAACAGGTTGTTACCCCCCAGGACACCATTGGACCAACGAGTGCACTTCTA

TTGCCCACTCAAGTTGAGACACCAAATGCTAGTGCACAGCGTGTGGAACTTGCTATGGCC

ACAGGGGCAGTGACCAGCAATGTGCCCAATTGCATCCGAGAGTGTTTTGCTGCGGTCACC

ACAATACCATGGACCACCCGGCAAGCAGCAAACACCTTTCTCGGTGCCATACATCTGGGC

CCACGCATAAA

>CMH-R140-18

ACTACCAAGTTAGTGTTTGAAATGGAGGGCTCGGGCCAGCCACAGTCCCAAAGGGACCAA

CAGGTTATGGAACAGGTTGTTACCCCCCAGGACACCATTGGACCAACGAGTGCACTTCTA

TTGCCCACTCAAGTTGAGACACCAAATGCTAGTGCACAGCGTGTGGAACTTGCTATGGCC

ACAGGGGCAGTGACCAGCAATGTGCCCAATTGCATCCGAGAGTGTTTTGCTGCGGTCACC

ACAATACCATGGACCACCCGGCAAGCAGCAAACACCTTTCTCGGTGCCATACATCTGGGC

CCACGCATAAA

>CMH-S175-18

ACTACCAAGTTAGTGTTTGAAATGGAGGGCTTGGGCCAGCCACAGTCCCAAAGGGACCAA

CAGGTTATGGAACAGGTTGTTACCCCCCAGGACACCATTGGACCAACGAGTGCACTTCTA

TTGCCCACTCAAGTTGAGACACCAAATGCTAGTGCACAGCGTGTGGAACTTGCTATGGCC

ACAGGGGCAGTGACCAGCAATGTGCCCAATTGCATCCGAGAGTGTTTTGCTGCGGTCACC

ACAATACCATGGACCACCCGGCAAGCAGCAAACACCTTTCTCGGTGCCATACATCTGGGC

CCACGCATAAA

>CMH-ST189-18

ACTACCAAGTTAGTGTTTGAAATGGAGGGCTTGGGCCAGCCACAGCCCCAAAGGGACCAA

CAGGTTATGGAACAGGTTGTCACTCCCCAGGACACCATTGGACCAACAAGTGCACTTCTA

TTGCCCACTCAAGTTGAGACACCAAATGCTAGTGCTCAGCGTGTGGAACTTGCAATGGCC

ACAGGGGCAGTGACCAGCAACGTGCCCAATTGCATCCGAGAGTGTTTTGCTGCGGTCACC

ACAATACCATGGACCACCCGGCAAGCAGCAAACACCTTTCTGGGTGCCATACATCTGGGA

CCACGCATCAA

>CMH-N145-12

AcTACCAAATTAGTGTTTGAAATGGAGGGCAATGCTCGCCCCGAGGGGCAAAACCCCGAG

CGCACGAATGTGCCACTGGCCTCCCCACAGGACACCATTGGCCCAAGTGCTGCGCTTTTG

CTCCCAACTCAAATTGAGACTCCAAACGCCACTGCACAGCGCGTGGAGCTGGCAGCTGCA

ACCGGAGCAATCACCAGCAATGTTCCGAGTTGCATTAGGGAGTGCTTTGCTAGCGTCACC

ACGCTCCCCTGGACCACTCGCCAAGCAGCCAACACGTTCTTGGGTGCCATCCATCTTGGA

CCGAGAATTAA

>CMH-N021-13

ACTACCaaATTAGTGTTTGAAATGGAGGGCAATGCTCGCCCCGAGGGGCAAAACCCCGAG

CGCTCAAATGTGCCATTGGCCTCCCCACAGGACACCATTGGCCCAAGTGCTACGCTCTTG

CTCCCAACTCAAATTGAAACTCCAAACGCCACTGCACAGCGTGTGGAGCTGGCAGCTGCA

ACCGGAGCAATCACCAGCAATGTTCCTAGTTGCATTAGGGAGTGCTTTGCCAGTGTCACT

ACGCTCCCCTGGACCACTCGCCAAGCAGCCAACACGTTCTTGGGTGCAATCCACCTTGGA

CCGAGGATCAA

>CMH-S050-17

ACTACCAAATTAGTGTTTGAAATGGAGGGCAATGCTCGCCCCGAAGGGCAAAACCCCGAG

CGCTCAAATGTGCCACTGGCCTCCCCACAGGACACCATTGGCCCAAGTGCTGCGCTCTTG

CTCCCAACTCAAATTGAAACTCCAAACGCCACTGCACAGCGCGTGGAGCTAGCAGCTGCA

ACTGGAGCAATCACCAGTAATGTTCCCAGTTGCATTAGGGAGTGCTTTGCCAGTGTCACC

ACGCTCCCCTGGACCACTCGCCAAGCAGCCAACACGTTCTTGGGTGCAATCCATCTTGGA

CCGAGGATCAa

>CMH-S057-17

ACTACCAAATTAGTGTTTGAAATGGAGGGCAATGCTCGCCCCGAAGGGCAAAACCCCGAG

CGCTCAAATGTGCCACTGGCCTCCCCACAGGACACCATTGGCCCAAGTGCTGCGCTCTTG

CTCCCAACTCAAATTGAAACTCCAAACGCCACTGCACAGCGCGTGGAGCTAGCAGCTGCA

ACTGGAGCAATCACCAGTAATGTTCCCAGTTGCATTAGGGAGTGCTTTGCCAGTGTCACC

ACGCTCCCCTGGACCACTCGCCAAGCAGCCAACACGTTCTTGGGTGCAATCCATCTTGGA

CCGAGGATCAA

>CMH-ST091-16

ACCACCAAATTAGTGTTTGAAATGGAGGGGCTTGGCTCCCAGCGAGGCCAGCGGCCTGAC

GAACAGGTTGCCAGCGATGCTGCTCAGGCCACCCCCCAAGATACAATTAGCCCCACAAGT

GCACTTTTGTTGCCAACTCAAATTGAGGTGCCAAACGCCGCAGCCCAGCGTGTTGAGTTA

GCTGCGGCAACAGGGGCCATTACCAGCAATGTCCCAAACTGTGTCCGTGAGTGCTTTGCT

GCTGTGACCACCATCCCATGGACCACACGGCAGGCTGCCAACACATTTTTGGGTGCCATC

CACCTTGGCCCACGCATCAA

>CMH-ST095-16

ACCACCAAATTAGTGTTTGAAATGGAGGGGCTTGGCTCCCAGCGAGGCCAGCGGCCTGAC

GAACAGGTTGCCAGCGATGCTGCTCAGGCCACCCCCCAAGATACAATTAGCCCCACAAGT

GCACTTTTGTTGCCAACTCAAATTGAGGTGCCAAACGCCGCAGCCCAGCGTGTTGAGTTA

GCTGCGGCAACAGGGGCCATTACCAGCAATGTCCCAAACTGTGTCCGTGAGTGCTTTGCT

GCTGTGACCACCATCCCATGGACCACACGGCAGGCTGCCAACACATTTTTGGGTGCCATC

CACCTTGGCCCACGCATCAA

>CMH-R089-18

ACCACCAAATTAGTGTTTGAAATGGAGGGACTCGGCTCCCAGCGAGGCCAGCAGCCTGAC

GAACAGGTTGCAAGCAATGCTGCACAGGCCGCTCCCCAAGACACAATCAGTCCCACGAGT

GCACTTTTGTTACCAACTCAAATTGAGATGCCAAACGCTGCTGCTCAGCGGGTTGAGTTG

GCCGCAGCAACAGGGGCCATAACCAGCAATGTCCCAAACTGTGTTCGTGAGTGCTTTGCT

TCTGTGACCACGATTCCATGGACCACGCGGCAGGCTGCCAACACGTTCCTGGGTGCCATT

CACCTTGGCCCACGCATCAA

>CMH-ST202-18

ACCACCAAATTAGTGTTTGAAATGGAGGGGCTTGGCTCCCAGCGAGGCCAGCGGCCTGAC

GAACAGGTTGCCAGCGATGCTGCTCAGGCCACCCCCCAAGATACAATCAGCCCCACAAGT

GCACTTTTGTTGCCAACTCAAATTGAGGTGCCAAACGCCGCAGCCCAGCGTGTTGAGTTG

GCTGCAGCAACAGGGGCTATTACTAGCAATGTCCCAAACTGTGTCCGTGAGTGTTTTGCT

GCCGTGACCACCATCCCATGGACCACGCGGCAGGCTGCCAACACGTTTTTGGGTGCCATC

CACCTTGGCCCTCGCATCAA

>CMH-ST207-18

ACCACCAAATTAGTGTTTGAAATGGAGGGGCTTGGCTCCCAGCGAGGCCAGCGGCCTGAC

GAACAGGTTGCCAGCGATGCTGCTCAGGCCACCCCCCAAGATACAATCAGCCCCACAAGT

GCACTTTTGTTGCCAACTCAAATTGAGGTGCCAAACGCCGCAGCCCAGCGTGTTGAGTTG

GCTGCAGCAACAGGGGCTATTACTAGCAATGTCCCAAACTGTGTCCGTGAGTGTTTTGCT

GCCGTGACCACCATCCCATGGACCACGCGGCAGGCTGCCAACACGTTTTTGGGTGCCATC

CACCTTGGCCCTCGCATCAA

>CMH-ST247-18

ACCACCAAATTAGTGTTTGAAATGGAGGGACTCGGCTCCCAGCGAGGCCAGCAGCCTGAC

GAACAGGTTGCAAGCAATGCTGCACAGGCCGCTCCCCAAGACACAATCAGTCCCACGAGT

GCACTTTTGTTACCAACTCAAATTGAGATGCCAAACGCTGCTGCTCAGCGGGTTGAGTTG

GCCGCAGCAACAGGGGCCATAACCAGCAATGTCCCAAACTGTGTTCGTGAGTGCTTTGCT

TCTGTGACCACGATTCCATGGACCACGCGGCAGGCTGCCAACACGTTCCTGGGTGCCATC

CACCTTGGCCCACGCATCAA

>CMH-ST266-18

ACCACCAAATTAGTGTTTGAAATGGAGGGACTCAGCTCCCAGCGAGGCCAACGGCCTGAT

GAACCGGTTGCCAGCAACGCTGCACAGGCCACTCCCCAAGACACAATCAGTCCCACGAGC

GCACTTTTGTTGCCAACTCAAATTGAGATGCCAAACGCTGCTGCCCAGCGTGTTGAGTTG

GCCGCAGCAACAGGGGCCATCACCAGCAACGTCCCAAACTGCGTTCGTGAGTGCTTTGCT

GCTGTGACTACGATTCCATGGACCACGCGGCAGGCTGCCAATACATTTCTGGGTGCTATT

CACCTTGGTCCACGTATCAA

>CMH-ST270-18

ACCACCAAATTAGTGTTTGAAATGGAGGGACTCGGCTCCCAGCGAGGCCAGCAGCCTGAC

GAACAGGTTGCAAGCAATGCTGCACAGGCCGCTCCCCAAGACACAATCAGTCCCACGAGT

GCACTTTTGTTACCAACTCAAATTGAGATGCCAAACGCTGCTGCTCAGCGGGTTGAGTTG

GCCGCAGCAACAGGGGCCATAACCAGCAATGTCCCAAACTGTGTTCGTGAGTGCTTTGCT

TCTGTGACCACGATTCCATGGACCACGCGGCAGGCTGCCAACACGTTCCTGGGTGCCATC

CACCTTGGCCCACGCATCAA

>CMH-N061-12

ACCACCAAATTAGTGTTTGAAATGGAGGGCAATGGCCTACCCCAGGCTGGACAACAGCAA

ACTCTCGACGTGCCAGGAACAACTGGCCCGACTTCGTCGGCAGTGGTGGTGGCAAATCCT

GACCAACCTTCTGCCCAGGCCCAACGCATGGAGCTGGCTGTTGCAACCGGTGCCGTGTCA

TCAAATGTCCCGGATGCAGTGCGTCAGTGCTTTGCACTCCTTCGCACATTTCCTTGGAAC

ACTCGACAGGCCACAGGCACTTATCTGGGATCTGCTGCGCTGTCCCCAGCTCTCAA

>CMH-N028-18

ACCACCAAATTAGTGTTTGAAATGGAGGGCAATGGCCTCCCTCAGGCTGGACAACAGCAA

GCTCTTGATGTGCCAGGAACAACTGGCCCGACTTCGTCGGCGGTGGTGGTGGCAAATCCT

GACCAACCTTCTGCCCAGGCCCAACGCATGGAACTGGCTGTTGCAACCGGTGCCGTGTCA

TCAAATGTCCCGGATGCAGTGCGTCAGTGCTTTGCACTCCTTCGCACATTTCCTTGGAAC

ACTCGACAGGCCACAGGCACTTATCTGGGATCTGCTGCGCTGTCCCCATCTCTCAA
